# Network localization of gray matter alterations in chronic smokers using the normative functional connectome

**DOI:** 10.3389/fpubh.2026.1762620

**Published:** 2026-03-27

**Authors:** Han Xu, Xiao-Yi Liu, Hu-Cheng Yang, Ping-Lei Pan, Si-Yu Gu, Wen-Hui Li, Shu Wang

**Affiliations:** 1 Department of Radiology, Affiliated Hospital 6 of Nantong University, Yancheng Third People’s Hospital, Yancheng, China; 2 Binhai Maternal and Child Health Hospital, Yancheng, China; 3 Department of Neurology, Affiliated Hospital 6 of Nantong University, Yancheng Third People’s Hospital, Yancheng, China; 4 Yancheng Maternal and Child Health Care Hospital, Yancheng, China

**Keywords:** basal ganglia, gray matter, network localization, Salience Network, smoking

## Abstract

**Background:**

Chronic smoking has well-documented impacts on brain structure. Voxel-based morphometry (VBM) investigations have revealed diverse regional gray matter (GM) changes in chronic smokers, hindering a unified understanding of smoking-induced neuropathology. To reconcile these findings, this study aimed to identify common intrinsic functional networks underlying these structural alterations using a functional connectivity network mapping (FCNM) approach. We further explored potential exposure-dependent variations to characterize how brain network architecture relates to cumulative smoking dose.

**Methods:**

We utilized coordinate-based FCNM to quantitatively integrate heterogeneous findings from previous VBM studies. We systematically reviewed VBM studies reporting GM differences between chronic smokers and non-smokers. We identified peak coordinates from 27 studies, encompassing 36 contrasts with 1,336 smokers and 1803 non-smokers. Resting-state fMRI from 1,093 healthy participants (Human Connectome Project) were utilized to create individual functional connectivity maps based on seed coordinates. Maps were combined to identify a shared alteration network and evaluated for spatial overlap with established canonical brain networks. Sensitivity analysis were conducted with different seed radii. Crucially, subgroup analysis stratified studies into higher-exposure and lower-exposure groups to investigate exposure-dependent mechanisms.

**Results:**

Functional connectivity network mapping identified a widespread network linked to smoking-induced GM changes. Key nodes included the supramarginal gyrus, insula, anterior cingulate cortex, caudate nucleus, putamen, and superior temporal gyrus. Spatial overlap analysis revealed predominant involvement of the posterior Salience Network (51.59%), anterior Salience Network (32.15%), basal ganglia network (31.52%), and auditory network (24.19%). Sensitivity analysis confirmed the robustness of these findings. Subgroup analysis revealed exposure-dependent patterns: while the Salience and basal ganglia networks were consistently affected in both groups, the auditory network and ventral Default Mode Network showed markedly greater involvement in the higher-exposure group, largely spared in the lower-exposure group.

**Conclusion:**

This FCNM approach identified consistent brain networks, predominantly the Salience, basal ganglia, and auditory networks, associated with chronic smoking-related GM alterations. These findings offer network-level insight into the structural effects of smoking, helping to resolve discrepancies and potentially guiding tailored interventions. Furthermore, the findings suggest a progressive neuropathological expansion, characterized by the concurrent recruitment of sensory (auditory) and high-order cognitive systems (ventral Default Mode Network) with cumulative smoking exposure.

## 1 Introduction

Tobacco use remains a major global public health threat, accounting for over 8 million deaths annually (1). Smoking is widely recognized for increasing the risk of cancer, respiratory diseases and cardiovascular diseases; Moreover, in addition to its adverse effects on oral, reproductive, and musculoskeletal health, smoking also causes profound damage to the nervous system (2–5). Nicotine, the primary addictive substance in cigarettes, binds to nicotinic acetylcholine receptors within key reward circuits, such as the ventral tegmental area and nucleus accumbens (6–10). This binding triggers the release of dopamine, generating a sense of pleasure and reinforcing the cycle of addiction (11). However, the regulatory effects of nicotine on dopamine in the brain are not limited to the reward circuit; nicotine may also influence other brain regions associated with mood, memory, and cognitive functions (11, 12). Other toxic compounds in cigarettes, such as carbon monoxide and free radicals, cause persistent neurological damage (e.g., cognitive decline) through mechanisms including oxidative stress, inflammation, vascular damage, and blood–brain barrier disruption (13, 14). Crucially, recent evidence links VBM-detected gray matter volume (GMV) changes directly to underlying cellular metrics, such as variations in cell density and nuclear volume (15). This suggests that smoking-induced oxidative and inflammatory burdens likely precipitate microscopic cellular alterations that manifest as macroscopic GM anomalies (16, 17). Although significant progress has been made in understanding the effects of smoking on the brain, the pathophysiology of brain changes in chronic smokers remains incompletely elucidated (18).

Over the past two decades, voxel-based morphometry (VBM) has been extensively used to examine gray matter (GM) volume alterations among chronic smokers (19–30). Compared with the manual delineation of regions of interest (ROI) for measuring brain structure volumes, VBM offers a hypothesis-free, time-efficient approach to quantifying whole-brain structural differences *in vivo* on a voxel-by-voxel basis (31). Taking advantage of this approach, numerous studies have found that chronic smokers exhibit reduced GM volume in regions such as the anterior cingulate cortex (ACC), thalamus, and cerebellum (19–24, 26, 28, 29). However, findings regarding GM volume changes in areas such as putamen, caudate nucleus and orbitofrontal cortex remain inconsistent (19–23, 26, 30, 32). Although this heterogeneity is often attributed to methodological variations, such as sample size, clinical characteristics, and analytical strategies (24, 33–37), divergent results persist even among well-controlled studies. In an attempt to synthesize these divergent VBM results, coordinate-based meta-analysis (CBMA) have been utilized to identify regions of spatial convergence, revealing consistent alterations in several regions, including the superior frontal gyrus, right lingual gyrus, ACC, left insula, left superior temporal gyrus, right anterior thalamic region and bilateral prefrontal cortex (PFC) (38–40). Nevertheless, CBMA is intrinsically limited by its focus on local spatial overlap. It treats each brain region as an independent unit, thereby overlooking the functional embedding of these regions within large-scale brain networks (41). Consequently, the persistent heterogeneity in VBM literature may not reflect random noise, but rather structural alterations in distinct nodes that share a common dysfunctional circuit.

The network-based perspective reframes the disparate regional findings as interconnected nodes within a large-scale dysfunctional circuit (42–44). This perspective is particularly compelling for chronic smoking, as converging evidence from resting-state functional Magnetic Resonance Imaging (rs-fMRI) studies have found altered functional connectivity (FC) within networks such as the Default Mode Network (DMN), Salience Network (SN), and Executive Control Network (ECN) in chronic smokers (45–47). Crucially, many of the regions encompassed by these networks—such as the ACC, dorsolateral PFC, and insula—are also the same areas repeatedly identified in VBM analysis, providing a strong indication of a structure–function correspondence (22, 23, 28). Indeed, beyond smoking, the broader field of addiction science has increasingly coalesced around the view of addiction as a circuit-level disorder, with neuroimaging evidence implicating disruptions in the SN, ECN, and reward networks across various substance use and behavioral addictions (48, 49). To rigorously test whether the heterogeneous GM alterations in smokers conform to this circuit-level hypothesis, methodological advancements such as functional connectivity network mapping (FCNM) offer a unique analytical framework. FCNM allows for the precise mapping of focal structural coordinates onto distributed brain networks, which may guide targeted clinical interventions (e.g., neuromodulation sites) (49–52). However, despite the strong theoretical rationale and the established utility of network mapping, a systematic FCNM-driven synthesis to reconcile the heterogeneous GM volume alterations reported in the chronic smoking literature has not yet been undertaken.

Therefore, to address the gap identified in the literature, the present study was designed with a threefold objective. First, we aimed to systematically characterize convergent regional GM alterations across prior whole-brain VBM studies of chronic smokers. Second, utilizing these localized peaks of structural alteration as seeds, we sought to identify the common functional brain networks associated with these changes. To achieve this, we employed a coordinate-based FCNM integrated with the large-scale Human Connectome Project (HCP) dataset. Third, to dissect the heterogeneity underlying these alterations, we performed subgroup analysis based on cumulative smoking exposure (pack-years) to elucidate potential dose-dependent trajectories of network dysregulation.

## 2 Materials and methods

### 2.1 Study search and selection

A comprehensive and systematic literature search was conducted across PubMed, Web of Science, and Embase to identify studies examining brain GM differences between chronic smokers and non-smokers published prior to September 1, 2025. The following search terms were used: (“smoking” OR “smokers” OR “nicotine” OR “tobacco” OR “cigarette”) AND (“voxel*” OR “VBM” OR “morphometry” OR “grey matter” OR “gray matter”). We also manually checked the reference lists of relevant reviews and meta-analysis to identify studies potentially missed by the database search.

The inclusion criteria were as follows: (1) original peer-reviewed articles published in English; (2) studies defining chronic smokers as individuals smoking at least five cigarettes daily; (3) studies employing VBM to compare GM differences between chronic smokers and non-smokers; (4) studies using significance thresholds corrected for multiple comparisons, or uncorrected thresholds with spatial extent correction; (5) studies reporting whole-brain VBM results as 3D coordinates (*x*, *y*, *z*) in Talairach or Montreal Neurological Institute (MNI) space.

The exclusion criteria were as follows: (1) pre-existing neuropsychiatric disorders (e.g., Parkinson’s disease, multiple sclerosis) or severe somatic disorders; (2) history of head injury with loss of consciousness, MRI contraindications, or a first-degree family history of psychiatric disorders. This study was conducted in accordance with the Preferred Reporting Items for Systematic Reviews and Meta-Analyses (PRISMA) 2020 guidelines, as illustrated in the selection flow diagram (Supplementary Figure S1) (53). Our analysis centered on the various comparisons within a single study, such as group comparisons between smokers and non-smokers in imaging patterns, rather than on the studies themselves.

Two independent reviewers extracted the peak coordinates (*x*, *y*, *z*) of significant gray matter volume differences between chronic smokers and non-smokers directly from the results tables or text of the included studies. We extracted all reported significant foci rather than selecting only the most significant one to maximize data coverage. To ensure spatial consistency across studies, coordinates originally reported in Talairach space were converted to MNI space using the Lancaster transform (icbm2tal) (54). This standardized set of MNI coordinates served as the input for the subsequent network mapping analysis.

### 2.2 Rs-fMRI dataset from HCP

This study utilized rs-fMRI data obtained from the HCP. The dataset included 1,093 healthy adults (594 females; mean age = 28.8, SD = 3.7 years), who were recruited at Washington University, primarily from families of twins and their non-twin siblings. To ensure data quality, participants with major radiological anomalies that could confound brain connectivity analysis were excluded. Participants with less severe focal abnormalities were not excluded but were documented with data flags on the HCP public wiki page (55). The age range for inclusion was limited to 22–35 years to enhance physiological and psychological stability in the sample, thus increasing the reliability and comparability of the results (55). Demographic information for the dataset is provided in Supplementary Table S1.

### 2.3 Rs-fMRI data acquisition and preprocessing

Rs-fMRI data from the HCP were collected on a 3 T Siemens Trio Scanner. The rs-fMRI acquisition parameters are detailed in Supplementary Table S2. Participants were excluded if their data exhibited poor image quality (e.g., visible artifacts) or incomplete brain coverage.

Rs-fMRI data preprocessing was performed utilizing Statistical Parametric Mapping 12 (SPM12; https://www.fil.ion.ucl.ac.uk/spm/) and the Data Processing & Analysis for Brain Imaging (DPABI; https://rfmri.org/DPABI) toolbox (56). The initial 10 volumes were excluded for each participant to achieve signal stability and participant acclimatization. The remaining volumes underwent slice-time correction and were then realigned to address head motion. The realignment process produced six motion parameters, comprising three translations and three rotations, for each volume. All participants’ data met the predetermined quality control standards for head motion, with maximal translation and rotation both below 2 mm and 2°, respectively. In order to enhance head motion control, framewise displacement (FD) was initially computed for each participant. A comprehensive nuisance regression was conducted subsequently to eliminate various confounding variables, including the linear trend, the 24 Friston motion parameters, signals from white matter and cerebrospinal fluid, and individual volumes flagged as motion outliers (FD > 0.5 mm). Global signal regression was incorporated in this step to enhance the detection of large-scale network correlations and to improve correspondence with structural connectivity (57). The datasets were subjected to bandpass filtering, preserving frequencies ranging from 0.01 to 0.1 Hz. The first stage of spatial normalization entailed co-registering the T1-weighted structural image of each participant with their average functional image. The structural images were transformed, segmented, and normalized to MNI space. Each filtered functional volume was then spatially normalized to MNI space. Finally, all functional data underwent spatial smoothing with a Gaussian kernel of 6 × 6 × 6 mm^3^ full-width at half maximum.

### 2.4 FCNM analysis

**Figure 1:**
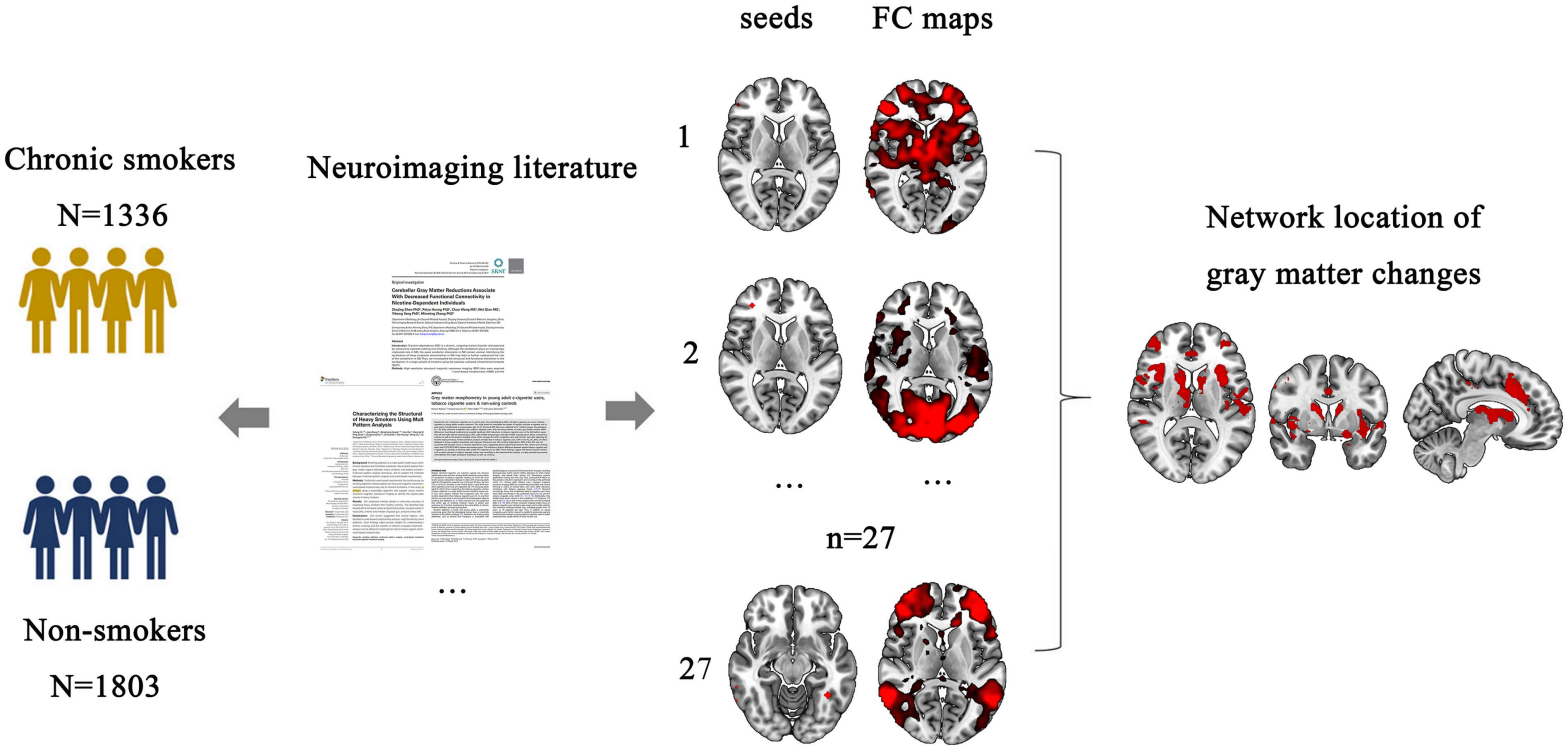
Study design and analysis pipeline. FC, functional connectivity.

To investigate brain networks linked to smoking-related structural GM volume alterations, we employed the FCNM approach. This method used coordinates of GM differences between chronic smokers and non-smokers as seeds. We delineated the functional network linked to smoking-induced GM changes using a five-step seed-based connectivity analysis implemented in MATLAB version 9.5 (R2018b). A schematic overview of the study design and analysis pipeline is provided in Figure 1. First, we formed a seed by creating 4-mm radius spheres centered on the peak coordinates identified in a previous structural analysis and consolidating them into a unified composite mask. Second, using rs-fMRI data from 1,093 HCP participants, individual seed-to-whole-brain FC maps were computed by calculating Pearson’s correlations between mean time course of the seed and every other brain voxel, followed by a Fisher’s z-transformation. Third, a group-level, one-sample t-test was conducted on the individual FC maps. This analysis focused solely on positive correlations due to the uncertain neurophysiological implications associated with negative FC (57, 58). Fourth, group t-maps were binarized at *p* < 0.05, following thresholding and correction for multiple comparisons using voxel-level false discovery rate. Finally, binarized FC maps were overlaid, and then thresholded at a 50% probability to create the ultimate smoking-related GM alteration network.

### 2.5 Association with canonical brain networks

To ascertain its functional significance, we mapped the smoking-related alteration network onto a canonical functional atlas comprising 14 established resting-state networks (59), thereby quantifying its spatial distribution across these systems. These canonical networks encompassed: anterior SN, left ECN (LECN), right ECN (RECN), basal ganglia network, ventral DMN (vDMN), language network, sensorimotor network, auditory network, posterior SN, visuospatial network, dorsal DMN (dDMN), precuneus network, high visual network, and primary visual network (59). The spatial association degree was determined by quantifying the percentage of voxels within each canonical network that showed spatial overlap with the smoking-related brain alteration network.

### 2.6 Subgroup analysis

To explore the potential heterogeneity of smoking-related network alterations and verify the robustness of our findings, we conducted subgroup analysis based on cumulative tobacco exposure. Studies were stratified into higher-exposure (*n* = 8) and lower-exposure (*n* = 8) smoking groups using the median pack-years as the cutoff value. Separate FCNM analysis were performed for each subgroup using the standard 4-mm radius. Within each subgroup, binarized FC maps were overlaid and applied a 60% threshold to define the consensus smoking-related GM alteration network. To further confirm the stability of these subgroup-specific findings, we also repeated the FCNM procedure using 1-mm and 7-mm radius spheres as sensitivity analysis.

## 3 Results

### 3.1 Included studies

Following a systematic literature search and selection process, our analysis incorporated 27 studies and 36 contrasts, representing data from 1,336 chronic smokers and 1803 non-smokers. There were no statistically significant differences between chronic smokers and non-smokers in average sample size (*p* = 0.22) or gender distribution (relative risk = 0.94, 95% CI = 0.87 to 1.02, *z* = −1.53, *p* = 0.127). Participants smoked an average of 17.32 ± 10.00 cigarettes per day, with a mean smoking history of 14.69 ± 11.99 pack-years. The average Fagerström Test for Nicotine Dependence (FTND) score was 4.96 ± 2.38, indicating moderate nicotine dependence. Other imaging characteristics of the included studies are summarized in Table 1.

**Table 1:** Demographic and clinical characteristics, and technical information of VBM studies included in the study.

Study	Sample (female)	Age (SD)	Smoking History (SD)	Cigarette/ day (SD)	Pack-years	FTND	Threshold	Software
Almeida et al. (2011) (164)	CS39 (25) NS39 (Na)	75.0 (3.4) 75.7 (3.2)	59 (Na)	25(Na)	Na	4 (Na)	*p* < 0.005 uncorrected	SPM2
Boparai et al. (2025) (165)	CS26 (9) NS25 (10)	22.15 (1.95) 22.36 (2.02)	5.23 (2.64)	8.24 (5.47)	Na	2.58 (2.18)	*p* < 0.05 corrected	SPM12
Brody et al. (2004) (111)	CS19 (11) NS17 (10)	39.5 (10.3) 37.9 (12.9)	31.0 (17.9)	26.2 (7.4)	Na	5.1 (1.9)	*p* < 0.001 uncorrected	SPM99
Bu et al. (2016) (22)	CS26 (0) NS26 (0)	21.42 (1.73) 20.58 (1.47)	4.27 (2.44)	15.04 (4.82)	3.55 (2.97)	4.42 (2.20)	*p* < 0.05 corrected	SPM8
Cai et al. (2022) (21)	CS23 (6) NS23 (6)	45.7 (6.8) 43.8 (9.4)	23.8 (7.9)	Na (Na)	Na	8.89 (0.71)	*p* < 0.05 corrected	SPM8
Chen et al. (2022) (166)	CS70 (29) NS209 (80)	29.79 (3.05) 29.54 (3.41)	15.14 (3.34)	Na	Na	4.83 (0.88)	*p* < 0.001 uncorrected	SPM
Conti et al. (2021) (20)	CS28 (10) NS24 (11)	28.1 (8.3) 28.5 (9.5)	Na	15.0 (4.5)	10.4 (8.1)	5.0 (1.5)	*p* < 0.05 corrected	SPM12
Daniju et al. (2022) (167)	CS19 (14) NS35 (20)	22.8 (3.6) 22.8 (4.9)	6.2 (4.2)	6.6 (5.3)	2.7 (3.65)	Na	*p* < 0.05 corrected	SPM12
Faulkner et al. (2021) (168)	CS12 (8) NS26 (15)	25.40 (4.58) 22.87 (4.60)	Na	11.45 (4.73)	6.21 (5.37)	Na	*p* < 0.001 corrected	SPM12
Franklin et al. (2014) (24)	CS80 (39) NS80 (39)	33.8 (Na) 22.1 (Na)	14.1 (Na)	14.7 (Na)	10.5 (Na)	4.45 (Na)	*p* < 0.025 corrected	SPM8
Fritz et al. (2014) (28)	CS315 (167) NS659 (416)	44.10 (11.84) 51.49 (14.45)	26.8 (Na)	13.17 (6.99)	17.81 (12.25)	Na	*p* < 0.05 corrected	SPM8
Gallinat et al. (2006) (26)	CS22 (12) NS23 (12)	30.8 (7.5) 30.3 (7.9)	13.9 (7.3)	14.5 (9.2)	13.5 (13.0)	2.9 (1.7)	*p* < 0.05 corrected	SPM2
Hanlon et al. (2016) (23)	CS58 (25) NS60 (27)	31.7 (Na) 29.7 (Na)	15.5 (Na)	16.2 (Na)	12.2 (Na)	4.6 (Na)	*p* < 0.01 corrected	SPM8
Kunas et al. (2020) (169)	CS62 (34) NS116 (67)	31.23 (9.5) 31.85 (10.8)	Na	Na	Na	Na	*p* < 0.001 uncorrected	SPM12
Liao et al. (2010) (170)	CS44 (36) NS44 (34)	28.1 (5.5) 26.3 (5.8)	10.4 (5.7)	20.3 (7.7)	Na	Na	*p* < 0.05 corrected	SPM5
Morales et al. (2012) (171)	CS25 (13) NS18 (8)	35.4 (1.8) 30.1 (2.2)	19 (Na)	14.1 (1.2)	11.5 (1.9)	3.8 (0.4)	*p* < 0.05 corrected	SPM8
Peng et al. (2015) (19)	CS26 (0) NS53 (0)	29.42 (4.43) 30.83 (5.18)	Na	16.15 (5.16)	8.77 (3.57)	Na	*p* < 0.05 corrected	SPM8
Peng et al. (2015) (19)	CS27 (0) NS53 (0)	32.26 (3.73) 30.83 (5.18)	Na	38.70 (8.36)	31.06 (7.40)	Na	*p* < 0.05 corrected	SPM8
Qian et al. (2019) (172)	CS44 (Na) NS41 (Na)	39 (6.5) 38.5 (7.4)	18.9 (6.4)	23.6 (10.4)	Na	5.4 (2.4)	*p* < 0.01 corrected	SPM8
Shen et al. (2018) (32)	CS85 (0) NS41 (0)	38.24 (6.81) 38.46 (8.60)	17.36 (6.58)	23.46 (9.53)	20.63 (12.28)	5.18 (2.18)	*p* < 0.05 corrected	SPM8
Stoeckel et al. (2016) (78)	CS16 (4) NS16 (5)	37.94 (11.61) 34.19 (7.20)	17.63 (10.49)	16.00 (4.84)	16.09 (12.17)	4.44 (2.16)	*p* < 0.05 corrected	SPM8
Wang et al. (2014) (25)	CS22 (0) NS20 (0)	22.48 (2.48) 21.80 (1.32)	4.95 (2.27)	11.90 (6.13)	3.10 (2.63)	Na	*p* < 0.05 corrected	SPM8
Weidler et al. (2024) (173)	CS52 (Na) NS45 (Na)	26.08 (6.91) 27.16 (6.15)	8.93 (7.89)	12.32 (4.48)	Na	3.72 (2.18)	*p* < 0.05 corrected	SPM
Weng et al. (2022) (174)	CS67 (Na) NS43 (Na)	29.22 (6.36) 29.51 (5.85)	Na	Na	Na	4.65 (1.76)	*p* < 0.05 corrected	SPM8
Ye et al. (2021) (175)	CS37 (8) NS28 (8)	47.18 (7.22) 43 (9.62)	25.34 (9.23)	35.13 (10.70)	Na	8.89 (0.68)	*p* < 0.05 corrected	SPM8
Yu et al. (2011) (29)	CS16 (Na) NS16 (Na)	41.6 (5.5) 39.2 (4.5)	21.1 (3.9)	20.6 (7.4)	Na	7.19 (1.42)	*p* < 0.05 corrected	SPM5
Zhang et al. (2022) (30)	CS28 (Na) NS28 (Na)	31.29 (5.56) 31.68 (6.57)	11.82 (5.77)	16.11 (8.35)	10.08 (7.95)	3.54 (2.03)	*p* < 0.001 corrected	SPM12
Zhang et al. (2011) (27)	CS48 (24) NS48 (24)	31.4 (8.1) 31.1 (8.8)	12.8 (7.4)	20.19 (6.6)	12.9 (7.9)	5.4 (1.9)	*p* < 0.05 corrected	FSL

VBM, voxel-based morphometry; Na, not available; FTND, Fagerstrom test of nicotine dependence; CS, chronic smokers; NS: non-smokers; SPM, statistical parametric mapping; FSL, functional magnetic resonance imaging of the brain software library.

### 3.2 Chronic smoking-related GM volume networks

**Figure 2:**
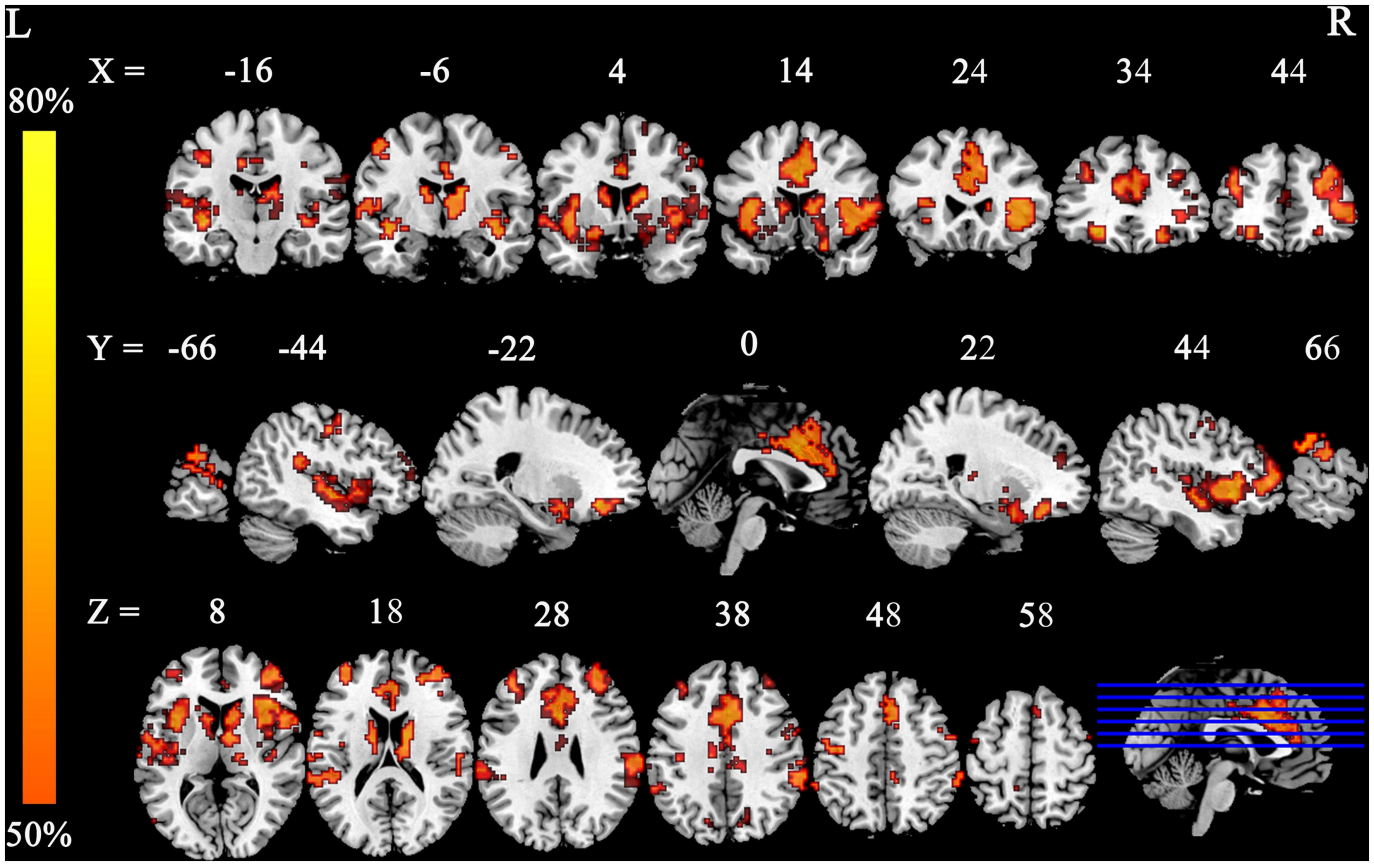
Smoking-related GM alteration networks based on 4 mm radius sphere. Smoking-related GM alteration networks are shown as network probability maps thresholded at 50%, showing brain regions functionally connected to more than 50% of the contrast seeds.

**Figure 3:**
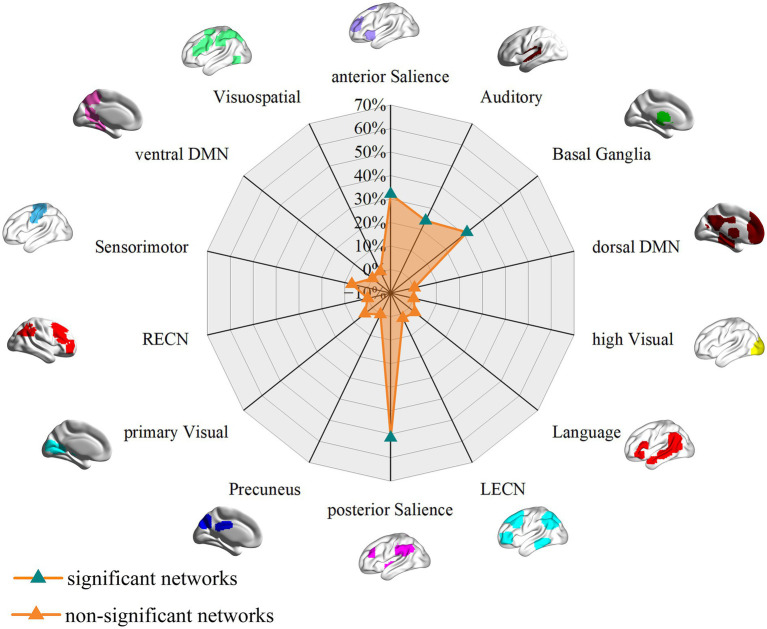
Associations of GM volume alteration networks with canonical brain networks in smokers based on 4 mm radius sphere. Polar plots display the proportion of overlapping voxels between each GM volume alteration network and a canonical network relative to all voxels within the corresponding canonical network. The green triangles represent GM volume alteration networks, defined as significant networks, exhibiting ≥ 10% overlap with canonical networks, whereas the orange triangles represent non-significant networks with <10% overlap.

The FCNM method revealed that the smoking-related GM alteration networks encompassed a distributed set of brain regions, prominently including the supramarginal gyrus (SMG), insula, ACC, caudate nucleus, putamen, and superior temporal gyrus (STG) (Figure 2). Spatial overlap analysis with canonical networks indicated that the chronic smoking-related network primarily involved the posterior SN (51.59%), anterior SN (32.15%), basal ganglia network (31.52%), and auditory network (24.19%) (Figure 3). Lower percentages of overlap were observed for other networks, including the LECN (1.85%), RECN (0%), vDMN (0%), language network (3.18%), sensorimotor network (6.98%), visuospatial network (0.21%), dDMN (0.37%), precuneus network (0.06%), high visual network (0%), and primary visual network (4.06%) (Figure 3). To confirm the robustness of these findings, we conducted sensitivity analysis by repeating the FCNM procedure with different seed radii. The brain alteration networks generated using 1-mm (Supplementary Figure S2) and 7-mm (Supplementary Figure S3) radius spheres were spatially highly similar to the network identified in the primary 4-mm analysis. Furthermore, a quantitative overlap analysis was performed for each sensitivity network. The analysis for the 1-mm radius network (Supplementary Figure S4) demonstrated that the posterior Salience, anterior Salience, basal ganglia, and auditory networks were the most predominantly involved systems. A highly consistent pattern was observed in the analysis for the 7-mm radius network (Supplementary Figure S5), which also identified these same networks as having the highest overlap. These results confirm the stability of our primary findings across different analytical parameters.

### 3.3 Subgroup analysis

**Figure 4:**
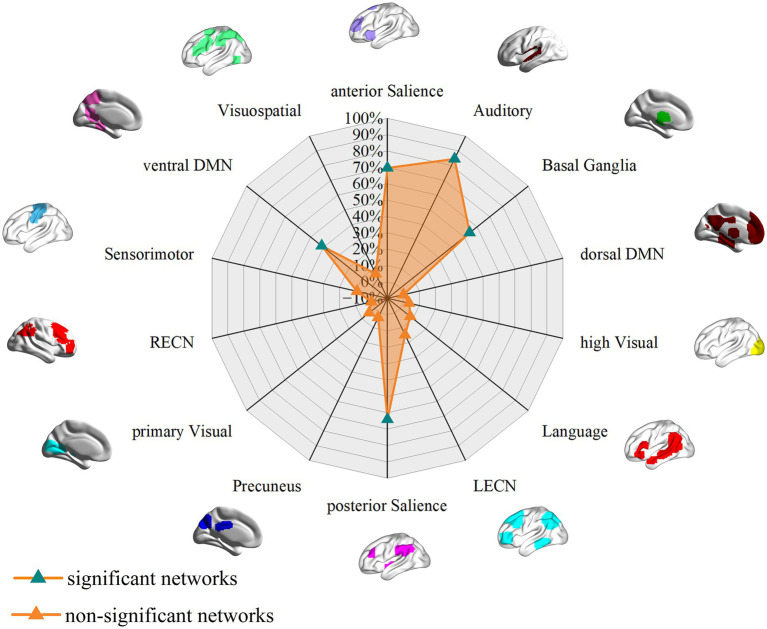
Associations of GM volume alteration networks with canonical brain networks in the higher-exposure subgroup based on a 4 mm radius sphere. Polar plots display the proportion of overlapping voxels between each GM volume alteration network and a canonical network relative to all voxels within the corresponding canonical network. The green triangles represent GM volume alteration networks, defined as significant networks, exhibiting ≥ 15% overlap with canonical networks, whereas the orange triangles represent non-significant networks with <15% overlap.

**Figure 5:**
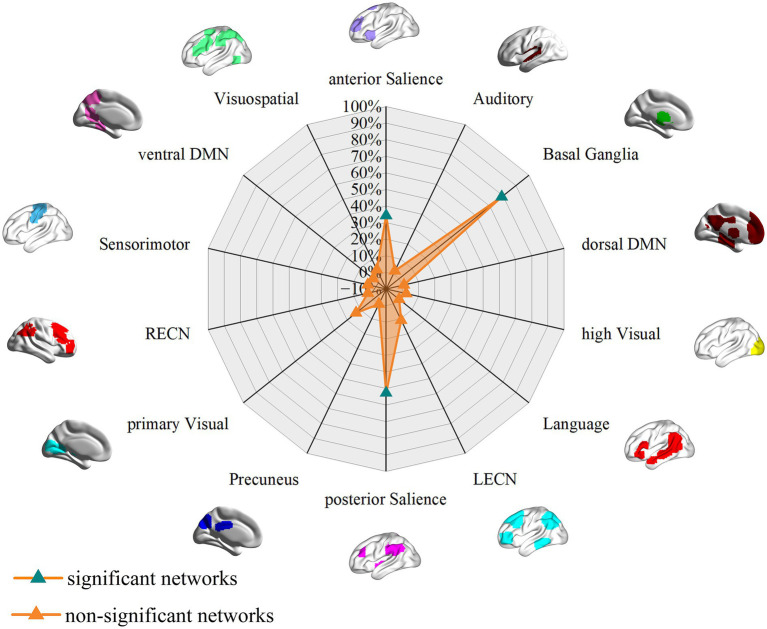
Associations of GM volume alteration networks with canonical brain networks in the lower-exposure subgroup based on a 4 mm radius sphere. Polar plots display the proportion of overlapping voxels between each GM volume alteration network and a canonical network relative to all voxels within the corresponding canonical network. The green triangles represent GM volume alteration networks, defined as significant networks, exhibiting ≥ 15% overlap with canonical networks, whereas the orange triangles represent non-significant networks with <15% overlap.

**Figure 6:**
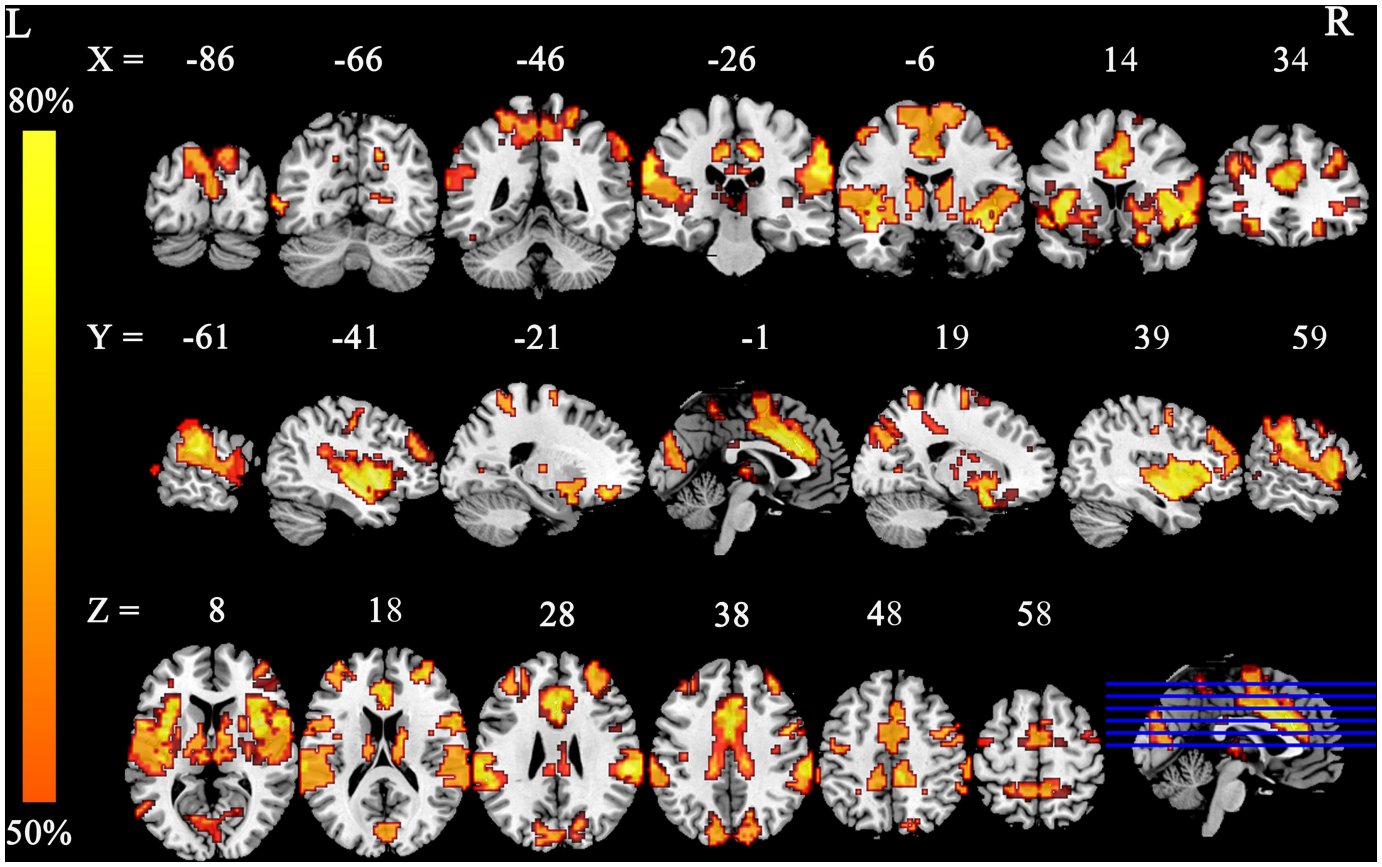
Smoking-related GM alteration networks in the higher-exposure subgroup based on a 4 mm radius sphere. Smoking-related GM alteration networks are shown as network probability maps thresholded at 60%, showing brain regions functionally connected to more than 60% of the contrast seeds.

**Figure 7:**
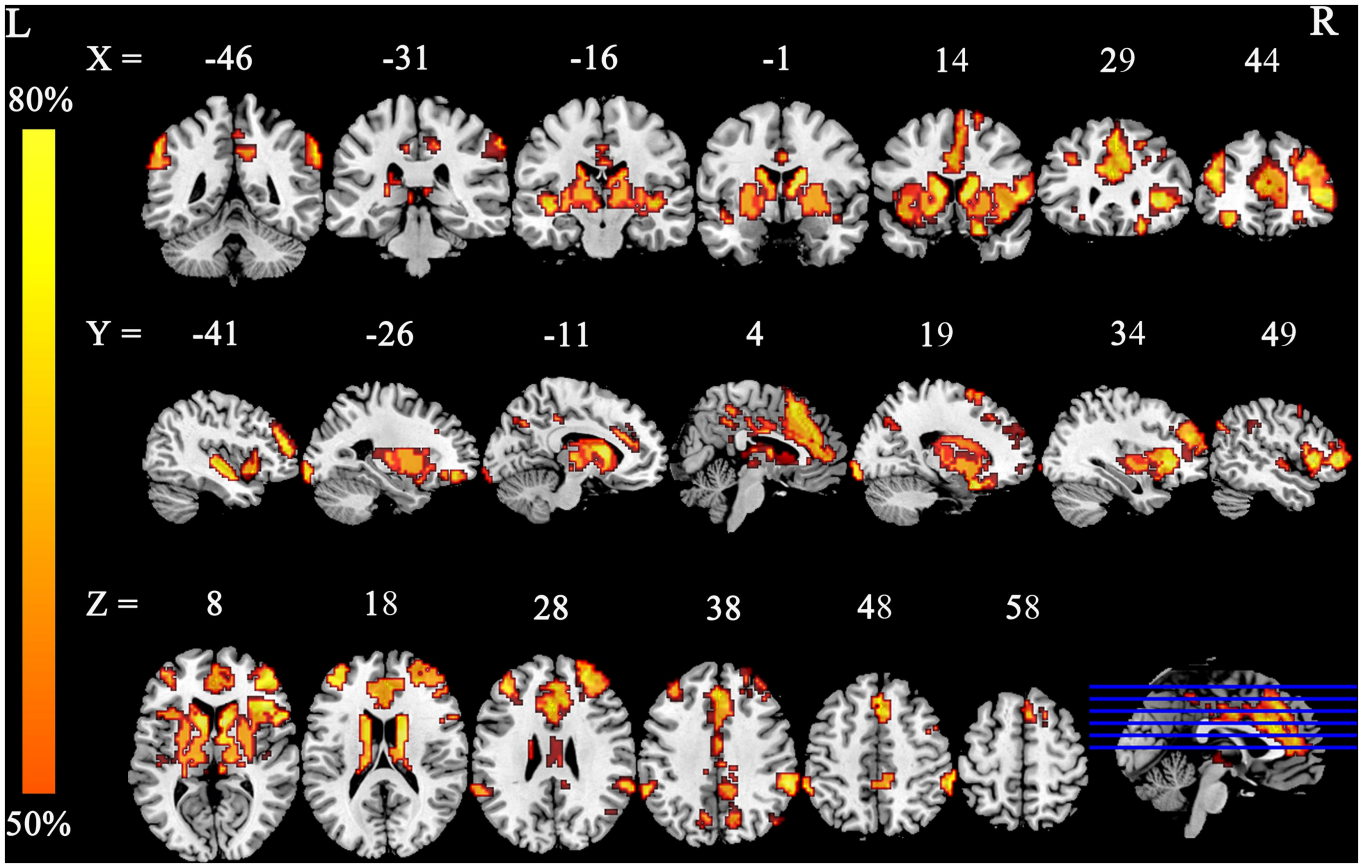
Smoking-related GM alteration networks in the lower-exposure subgroup based on a 4 mm radius sphere. Smoking-related GM alteration networks are shown as network probability maps thresholded at 60%, showing brain regions functionally connected to more than 60% of the contrast seeds.

To investigate the impact of cumulative tobacco exposure on network alterations, we stratified the included studies reporting pack-year information into higher-exposure (*n* = 8) and lower-exposure (*n* = 8) subgroups using the median value as the cutoff. Detailed characteristics of the included studies for each subgroup are provided in Supplementary Tables S3, S4. FCNM analysis revealed that both subgroups exhibited alteration patterns largely overlapping with the main findings, particularly involving the posterior and anterior SN and the basal ganglia network (Figures 4, 5). However, notable differences were observed the higher-exposure subgroup demonstrated more extensive involvement of the vDMN (Figure 4), whereas the auditory network showed negligible alterations in the lower-exposure subgroup (Figure 5). The specific anatomical regions corresponding to these distinct network patterns are detailed in Figures 6, 7. To verify the robustness of these subgroup-specific findings, we conducted sensitivity analysis by repeating the FCNM procedure using 1-mm (Supplementary Figures S6–S9) and 7-mm (Supplementary Figures S10–S13) radius spheres. The resulting network patterns were highly consistent with those identified using the primary 4-mm radius, confirming the stability of our subgroup results across different analytical parameters. These results suggest a potential dose-dependent effect of smoking on brain network integrity.

## 4 Discussion

This study utilized the FCNM approach and a large-scale human connectome dataset to elucidate smoking-related GM alterations at the network level. The identified smoking-associated GM alteration networks encompassed spatially distributed brain regions, predominantly involving the posterior SN (pSN), anterior SN (aSN), basal ganglia network, and auditory network. The robustness of the FCNM was demonstrated by reproducing it with varying radii (1-mm and 7-mm). These findings provide empirical evidence for network-level GM alterations in chronic smokers, enhancing our understanding of the neuropathophysiological mechanisms of smoking addiction from a network perspective and potentially guiding the development of targeted interventions for it.

### 4.1 Abnormal pSN and aSN in smokers

The SN, particularly its posterior component, exhibited significant disruption in the smokers identified in our study. The pSN, including the SMG identified as an important node in our study, plays a crucial role in task switching, attention distribution, and the coordination of behavioral control (60–62). Consistent with the disruption, rs-fMRI analysis has shown a trend of decreased FC in the pSN of chronic smokers compared to non-smokers, a deficit often associated with cognitive impairment (63, 64). The aSN was also found to be involved in the network of GM alterations associated with smoking. This network, centered on the anterior insula and dorsal ACC, is vital for salience detection, executive functions, and emotional regulation (65–68). Our findings align with rs-fMRI studies that have reported reduced FC within the aSN in smokers, a finding that has also been linked to severity of nicotine addiction (69–72).

The SMG, identified as a critical node of pSN, displayed noteworthy structural and functional changes of chronic smokers in recent studies (29, 73–75). Reduced FC was noted between the SMG and prefrontal regions (76), alongside sex-specific connectivity patterns associated with the basal nucleus of Meynert (74). Moreover, smokers exhibited notably reduced regional cortical perfusion in the right SMG compared to non-smokers (77).

Within the aSN, converging evidence from VBM studies consistently indicates altered GM volume and density in the anterior insula of smokers (19, 25–27), which is negatively correlated with daily cigarette consumption (78). Crucially, damage to the anterior insula diminishes cravings, providing compelling evidence for its causal role in addiction (79). In line with this structural and causal evidence, rs-fMRI unequivocally demonstrates altered anterior insula function, manifesting as heightened reactivity to negative stimuli, particularly in heavier smokers, and weakened anterior insula-ACC connectivity associated with dependence (80–84). The anterior insula, being a central hub in the triple network (SN-central executive network-DMN) and exerting significant influence over impulse control and craving, emerges as a key candidate for therapeutic interventions (85–90). Similarly, the dorsal ACC, a key partner of the anterior insula within the aSN (65), also displays marked abnormalities in chronic smokers. Event-related potential and VBM studies converge to reveal inhibitory control deficits and reduced GM volume in the dorsal ACC (21, 22, 28, 91). Moreover, fMRI-based neurofeedback and abstinence studies have demonstrated significant disruptions in ACC activity and connectivity patterns in chronic smokers, which are associated with craving, cognitive impairments, and heightened risk of relapse (92–95). Notably, interventions such as integrative body–mind training and mindfulness-based therapies have the potential to regulate dorsal ACC activity, thereby enhancing smoking cessation results (96, 97), thus solidifying the position of dorsal ACC as a crucial therapeutic target within the SN (98).

### 4.2 Abnormal basal ganglia network in smokers

The basal ganglia network is acknowledged for its essential involvement in various functions, encompassing motor learning, executive functions, behavioral regulation, and emotional processing (99). Crucially, it is central to reward processing and plays a significant role in smoking addiction (100–102). Converging evidence from multiple neuroimaging modalities reveals the underlying mechanisms at different levels (80, 103–105). First, at the neurochemical level, positron emission tomography (PET) studies have demonstrated that smoking disrupts dopamine activity within the basal ganglia network, leading to a cascade of neural adaptations that encompass altered reward responses, activation of stress-related systems, and impaired cognitive control, all of which contribute to the maintenance and intensification of nicotine addiction (103). Second, rs-fMRI studies consistently show reduced FC between basal ganglia regions (such as the caudate and putamen) and other brain areas (80). Furthermore, dynamic FC analysis reveal that this network dysfunction is not static; instead, it involves dynamic changes that heighten the sensitivity of the reward system, thereby promoting the maintenance of smoking behavior (104, 105). Together, these findings firmly establish the basal ganglia network as a critical hub in addictive smoking behaviors.

As core components of the basal ganglia network, the striatum (primarily the caudate nucleus and putamen) plays a pivotal role in cognition, learning, and motivation, and its dysfunction has been consistently implicated in smoking addiction (76, 106, 107). Converging neuroimaging evidence reveals widespread functional abnormalities within the striatum of smokers. For instance, functional Magnetic Resonance Imaging (fMRI) and PET studies have identified reduced FC and diminished cerebral blood flow in the caudate nucleus (76, 108, 109). Similarly, the putamen exhibits altered FC with regions like the insula, alongside dopamine-related abnormalities (110, 111). Underlying these functional alterations is a profound neurochemical imbalance. Research shows that dopamine dysregulation in the striatum reinforces nicotine addiction (80, 111, 112), whereas modulating striatal dopamine and FC of striatum may facilitate nicotine withdrawal. This not only highlights the importance of striatum in the addiction mechanism but also underscores its potential as a therapeutic target. Therefore, targeting the striatum and the broader basal ganglia network with interventions such as deep brain stimulation or repetitive transcranial magnetic stimulation shows significant promise, offering a key direction for the development of effective smoking cessation therapies (100, 104, 113–115).

### 4.3 Abnormal auditory network in smokers

While the roles of the pSN, aSN, and basal ganglia network in smoking-related neurobiological changes are well-documented, research on the auditory network remains limited. The lack of research on this topic raises important questions about its potential role in the neurobiology of smoking behavior, possibly influencing cue-induced reactivity or sensory integration within the auditory network (116, 117).

The auditory network is mainly tasked with processing auditory information and associated cognitive functions, thereby playing a crucial role in environmental interactions (118, 119). Despite its lesser established role in core addiction processes like reward when compared to the basal ganglia network, the auditory system may potentially influence smoking behavior through pathways related to cue reactivity or altered sensory processing, in conjunction with recognized clinical connections between smoking and hearing impairment (116, 117, 120–122). Neuroimaging evidence increasingly suggests structural and functional alterations in the auditory network, particularly within the STG, among chronic smokers (19, 26, 123, 124).

The STG is primarily associated with auditory processing and cross-modal integration (125–127). Structural investigations in smokers consistently reveal abnormalities in this region, characterized by altered GM volume/density and cortical thinning in the STG (19, 26, 128–130). Altered intrinsic activity in the STG and decreased FC have been observed in complementary rs-fMRI studies, which are associated with nicotine intake, severity of dependence, relapse, impulsivity, and impaired control (123, 124). Task-fMRI showed heightened STG reactivity to smoking cues (131–133), and arterial spin labeling MRI demonstrated reduced STG perfusion in smokers (77). Taken together, these findings highlight the auditory network, particularly the STG, as being affected by chronic smoking. The auditory network alteration may serve as a neural marker and potential predictor of cessation outcomes (123). Despite historical under-emphasis, considering the auditory network is crucial for future smoking research and intervention development.

### 4.4 Subgroup analysis

Our subgroup analysis stratified by pack-years provided critical insights into the potential dose-dependent nature of smoking-related network alterations. First, the SN and basal ganglia network were consistently identified in both higher- and lower-exposure subgroups. This suggests that structural vulnerabilities in these systems—which are important for attention distribution, emotional processing and reward processing——represent the core neuropathology of nicotine addiction (60–62, 99–102). These alterations likely emerge relatively early in the addiction trajectory and persist throughout the course of the disorder. Second, a notable divergence was observed in the vDMN, characterized by extensive involvement specifically within the higher-exposure group. While the DMN is primarily implicated in self-referential processing and episodic memory (134), recent literature indicates that greater nicotine dosage and duration drive significant functional and structural alterations within this network, including altered regional homogeneity and efficiency (75, 130, 135–137). Crucially, within this dysregulated vDMN, the Inferior Parietal Lobule (IPL) may serve as a pivotal hub particularly vulnerable to these exposure-related alterations. As a heteromodal association hub integrating sensory inputs with internal memory representations (138, 139), the IPL in smokers shows exposure-related cortical thinning and heterogeneous functional-dynamic alterations with the insula (137, 140–142). Collectively, these findings suggest a hemispheric lateralization of control failure: reduced dynamic flexibility in right frontoparietal regions creates a neural shortcut where smoking cues bypass executive oversight, directly activating automated approach tendencies (61, 142, 143). This transition from goal-directed to habitual control is evidenced by reduced effective connectivity from parietal nodes to the medial PFC, functionally disconnecting executive oversight and allowing drug cues to bypass conscious deliberation (135, 144–146). This mechanism may help explain why higher pack-years are associated with greater behavioral automaticity in smoking: we hypothesize that the IPL serves as a sensitized gateway that preferentially processes nicotine-associated stimuli. Furthermore, Hyper-synchrony of the posterior DMN and impaired switching to the executive control network likely underlie attentional bias and cognitive inflexibility in chronic smokers (147, 148). Consequently, therapeutic interventions such as repetitive transcranial magnetic stimulation and mindfulness-based neurofeedback should specifically targeting DMN nodes including the IPL to downregulate DMN hyperconnectivity of chronic smokers and restore the balance between internal rumination and external attention (145, 149–151). Third, the auditory network showed negligible structural alterations in the lower-exposure group, despite being prominent in the main analysis and higher-exposure group. This implies that structural deficits in primary sensory processing regions might be a cumulative, late-stage consequence of chronic neurotoxicity or vascular damage associated with long-term smoking, rather than a predisposing factor for addiction (130). Finally, the high spatial consistency of these subgroup-specific patterns across different seed radii (1-mm, 4-mm, and 7-mm) reinforces the robustness of these findings, suggesting that the observed exposure-dependent distinctions reflect genuine biological heterogeneity rather than methodological artifacts.

### 4.5 Limitations and future perspectives

This study has several limitations that should be considered when interpreting the findings. First, the analysis depends on coordinate-based data extracted from published studies, which provides a summary statistic (peak location) and inherently loses spatial information compared to analysis using full statistical maps or individual participant data (50, 152–154). This coordinate-based approach is correlational and thus cannot establish causality between the identified network and smoking-related GM alterations (43). Future work integrating causal data, such as lesion network mapping studies relevant to addiction (49), could strengthen the interpretation. Second, considerable heterogeneity is observed among the included studies in terms of sample characteristics (e.g., smoking duration, severity, and demographics), imaging acquisition parameters, preprocessing pipelines, and statistical approaches (24, 155). Although FCNM aims to identify convergence amidst variability, the noise and biases introduced by this heterogeneity can obscure the discovery of a common network (50, 152). Furthermore, our FCNM analysis did not weight studies by sample size or effect size, as there is currently no universally accepted method for incorporating these criteria. Third, we utilized a normative connectome derived from healthy adults (HCP dataset) to map the networks associated with smoking-related GM alterations. This approach aligns with standard network mapping practices, where evidence indicates that sample selection has minimal impact on network localization (43, 50, 153, 156–159). However, this method may introduce bias or reduce precision due to age or ethnic differences between the populations. Although a smoking-specific connectome might yield slight variations, research suggests such differences are negligible (49). Fourth, the study’s retrospective design necessitates prospective validation of the identified smoking-related network in independent cohorts of smokers and non-smokers. Additionally, our exclusion of individuals who had quit smoking constrained our ability to assess the reversibility of the identified network alterations following cessation. Fifth, methodological choices, such as the radius of the coordinate spheres (although sensitivity analysis demonstrated robustness) and the probability threshold (set at 50%) used to define the final network, are important parameters that can influence the results (50, 160). The exploration of alternative parameters for additional insights was not within the scope of this initial network localization. Sixth, we identified a network linked to GM alterations in smokers; however, we did not conduct formal specificity testing to compare this network with those derived from coordinates associated with other conditions, such as other substance use disorders, aging, or other psychiatric conditions, as performed in certain network mapping studies (152, 160). Such comparisons would enhance assertions regarding the distinctiveness of the identified network in relation to smoking. Seventh, the subgroup analysis regarding smoking severity were constrained by the limited availability of detailed pack-year data in the included studies. Although we successfully stratified a subset of studies to explore dose-dependent effects, this classification relied on the reported mean pack-years for each study cohort. Consequently, this aggregate measure masks intra-sample heterogeneity, as individual participants within the same study likely exhibited wide variations in exposure levels above or below the mean. Furthermore, the reduced sample size in each subgroup (n = 8) may limit statistical power compared to the main analysis. Additionally, the potential collinearity between age and cumulative smoking exposure (pack-years) poses a challenge in fully disentangling their independent contributions to network alterations (161). Future studies utilizing individual-level data with large samples and prospective longitudinal designs are needed to precisely dissociate the effects of aging from chronic nicotine toxicity (162, 163). Finally, our findings should be viewed within the context of the persistent challenges affecting reproducibility in neuroimaging, including small sample sizes, clinical heterogeneity, experimental variability, and analytical flexibility (24, 34, 37). Continued efforts towards standardization, larger sample sizes, and transparent reporting are crucial for advancing the field.

## 5 Conclusion

In conclusion, this study adeptly applied the FCNM method, utilizing human connectome data, to synthesize the findings from previous VBM studies of chronic smokers that reported heterogeneous neuroanatomical changes. We identified consistent brain networks associated with smoking-related GM alterations, primarily encompassing regions within the pSN/aSN, basal ganglia network, and auditory network. Crucially, our subgroup analysis revealed an exposure-dependent expansion of neuropathology: while core control systems (Salience and basal ganglia networks) are consistently affected, structural deficits progressively recruit the auditory network and vDMN in individuals with higher cumulative smoking exposure. These findings provide robust, network-level evidence for the structural impact of chronic smoking on the brain. By demonstrating that seemingly disparate regional findings converge onto specific functional networks, this work helps reconcile inconsistencies in the prior literature and significantly extends our understanding of the neuropathology of smoking from a systems perspective. The identification of these smoking-related networks provides valuable insights that could inform the development of more targeted and effective smoking cessation programs and prevention strategies.

## Data Availability

The original contributions presented in the study are included in the article/Supplementary material, further inquiries can be directed to the corresponding authors.

## Supplementary material

SUPPLEMENTARY FIGURE S1 Flow diagram of study identification and selection in accordance with PRISMA 2020 guidelines. GM, gray matter; ROI, region of interest; VBM, voxel-based morphometry.

SUPPLEMENTARY FIGURE S2 Smoking-related GM alteration networks based on 1-mm radius sphere. Smoking-related GM alteration networks are shown as network probability maps thresholded at 50%, showing brain regions functionally connected to more than 50% of the contrast seeds.

SUPPLEMENTARY FIGURE S3 Smoking-related GM alteration networks based on 7-mm radius sphere. Smoking-related GM alteration networks are shown as network probability maps thresholded at 50%, showing brain regions functionally connected to more than 50% of the contrast seeds.

SUPPLEMENTARY FIGURE S4 Associations of GM volume alteration networks with canonical brain networks in smokers based on 1-mm radius sphere. Polar plots display the proportion of overlapping voxels between each GM volume alteration network and a canonical network relative to all voxels within the corresponding canonical network. The green triangles represent GM volume alteration networks, defined as significant networks, exhibiting ≥ 10% overlap with canonical networks, whereas the orange triangles represent non-significant networks with <10% overlap.

SUPPLEMENTARY FIGURE S5 Associations of GM volume alteration networks with canonical brain networks in smokers based on 7-mm radius sphere. Polar plots display the proportion of overlapping voxels between each GM volume alteration network and a canonical network relative to all voxels within the corresponding canonical network. The green triangles represent GM volume alteration networks, defined as significant networks, exhibiting ≥ 10% overlap with canonical networks, whereas the orange triangles represent non-significant networks with <10% overlap.

SUPPLEMENTARY FIGURE S6 Smoking-related GM alteration networks in the higher-exposure subgroup based on a 1-mm radius sphere. Smoking-related GM alteration networks are shown as network probability maps thresholded at 60%, showing brain regions functionally connected to more than 60% of the contrast seeds.

SUPPLEMENTARY FIGURE S7 Associations of GM volume alteration networks with canonical brain networks in the higher-exposure subgroup based on a 1-mm radius sphere. Polar plots display the proportion of overlapping voxels between each GM volume alteration network and a canonical network relative to all voxels within the corresponding canonical network. The green triangles represent GM volume alteration networks, defined as significant networks, exhibiting ≥ 22% overlap with canonical networks, whereas the orange triangles represent non-significant networks with <22% overlap.

SUPPLEMENTARY FIGURE S8 Smoking-related GM alteration networks in the lower-exposure subgroup based on a 1-mm radius sphere. Smoking-related GM alteration networks are shown as network probability maps thresholded at 60%, showing brain regions functionally connected to more than 60% of the contrast seeds.

SUPPLEMENTARY FIGURE S9 Associations of GM volume alteration networks with canonical brain networks in the lower-exposure subgroup based on a 1-mm radius sphere. Polar plots display the proportion of overlapping voxels between each GM volume alteration network and a canonical network relative to all voxels within the corresponding canonical network. The green triangles represent GM volume alteration networks, defined as significant networks, exhibiting ≥ 15% overlap with canonical networks, whereas the orange triangles represent non-significant networks with <15% overlap.

SUPPLEMENTARY FIGURE S10 Smoking-related GM alteration networks in the higher-exposure subgroup based on a 7-mm radius sphere. Smoking-related GM alteration networks are shown as network probability maps thresholded at 60%, showing brain regions functionally connected to more than 60% of the contrast seeds.

SUPPLEMENTARY FIGURE S11 Associations of GM volume alteration networks with canonical brain networks in the higher-exposure subgroup based on a 7-mm radius sphere. Polar plots display the proportion of overlapping voxels between each GM volume alteration network and a canonical network relative to all voxels within the corresponding canonical network. The green triangles represent GM volume alteration networks, defined as significant networks, exhibiting ≥ 22% overlap with canonical networks, whereas the orange triangles represent non-significant networks with <22% overlap.

SUPPLEMENTARY FIGURE S12 Smoking-related GM alteration networks in the lower-exposure subgroup based on a 7-mm radius sphere. Smoking-related GM alteration networks are shown as network probability maps thresholded at 60%, showing brain regions functionally connected to more than 60% of the contrast seeds.

SUPPLEMENTARY FIGURE S13 Associations of GM volume alteration networks with canonical brain networks in the lower-exposure subgroup based on a 7-mm radius sphere. Polar plots display the proportion of overlapping voxels between each GM volume alteration network and a canonical network relative to all voxels within the corresponding canonical network. The green triangles represent GM volume alteration networks, defined as significant networks, exhibiting ≥ 15% overlap with canonical networks, whereas the orange triangles represent non-significant networks with <15% overlap.

## Glossary

ACC: anterior cingulate cortex
AI: artificial intelligence
aSN: anterior Salience Network
CI: confidence interval
CS: chronic smokers
dDMN: dorsal Default Mode Network
DMN: Default Mode Network;
DPABI: data processing & analysis for brain imaging
F: female
FA, flip angle
FC: functional connectivity
FCNM: functional connectivity network mapping
FD: framewise displacement
fMRI: functional Magnetic Resonance Imaging
FOV: field of view
FSL: functional magnetic resonance imaging of the brain software library
FTND: Fagerstrom test of nicotine dependence
GM: gray matter
GRE-EPI: gradient-recalled echo-Planar Imaging
HCP: Human Connectome Project
IPL: Inferior Parietal Lobule
LECN: left executive control network
M: male
MNI: Montreal Neurological Institute
MRI: Magnetic Resonance Imaging
ms: millisecond
Na: not available
NS: non-smokers
p: *p*-value
PET: positron emission tomography
PFC: prefrontal cortex
PRISMA: Preferred Reporting Items for Systematic Reviews and Meta-Analyses
pSN: posterior Salience Network
RECN: right executive control network
ROI: region of interest
rs-fMRI: resting-state functional Magnetic Resonance Imaging
SN: Salience Network
SD, standard deviation
SDM: seed-based d mapping
SMG: supramarginal gyrus
SPM: Statistical Parametric Mapping
STG: superior temporal gyrus
TE: echo time
TR: repetition time
VBM: voxel-based morphometry
vDMN: ventral default mode network
z: *z*-score
°: degrees
