# Supplementary figures and images for: Network localization of gray matter alterations in chronic smokers using the normative functional connectome
Source: Front Public Health. 2026 Mar 27;14:1762620. doi: 10.3389/fpubh.2026.1762620 (PMC13066286; doi:10.3389/fpubh.2026.1762620)

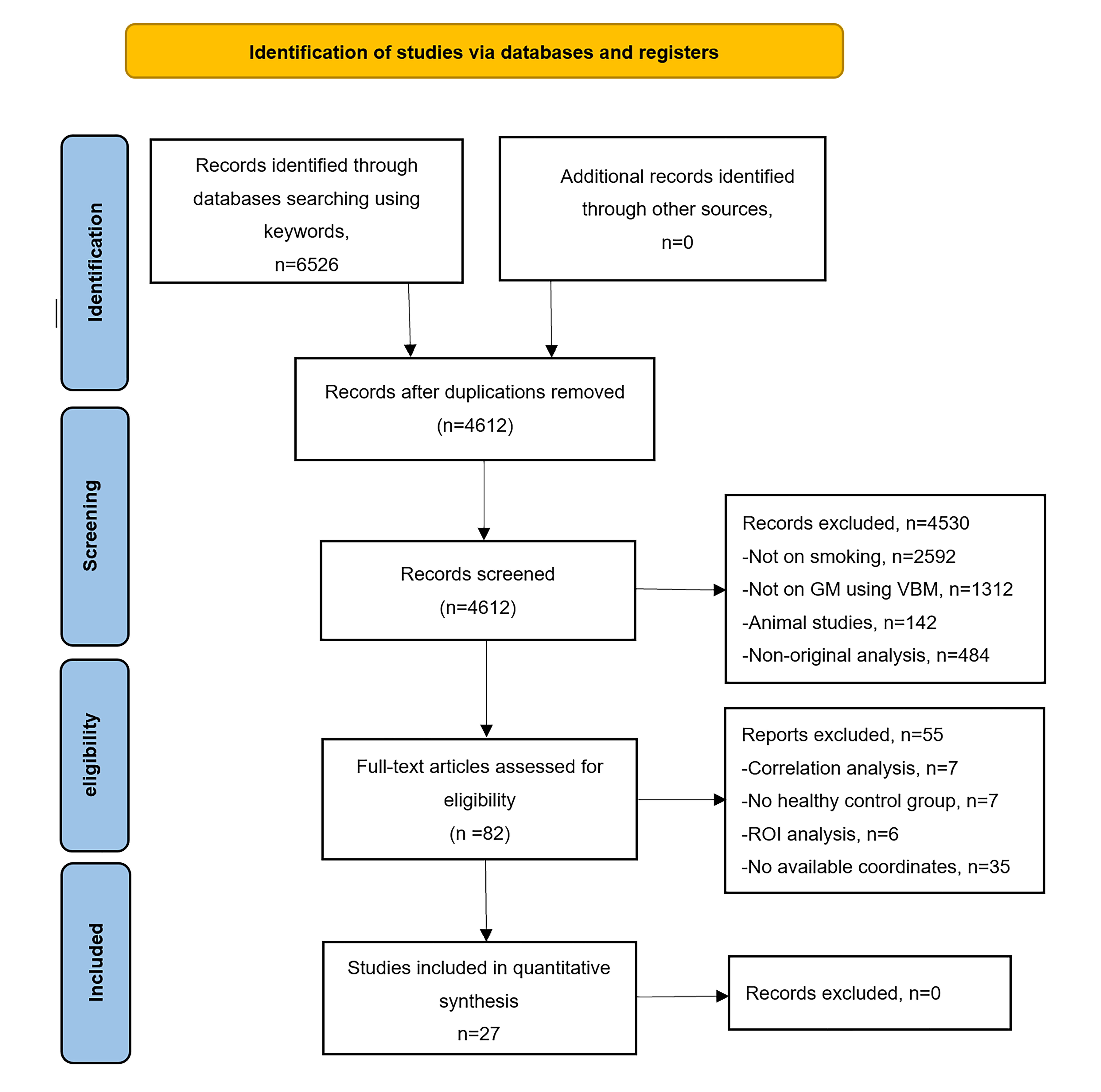

Supplement: SUPPLEMENTARY FIGURE S1 — Flow diagram of study identification and selection in accordance with PRISMA 2020 guidelines. GM, gray matter; ROI, region of interest; VBM, voxel-based morphometry. [file Image_1.tif]

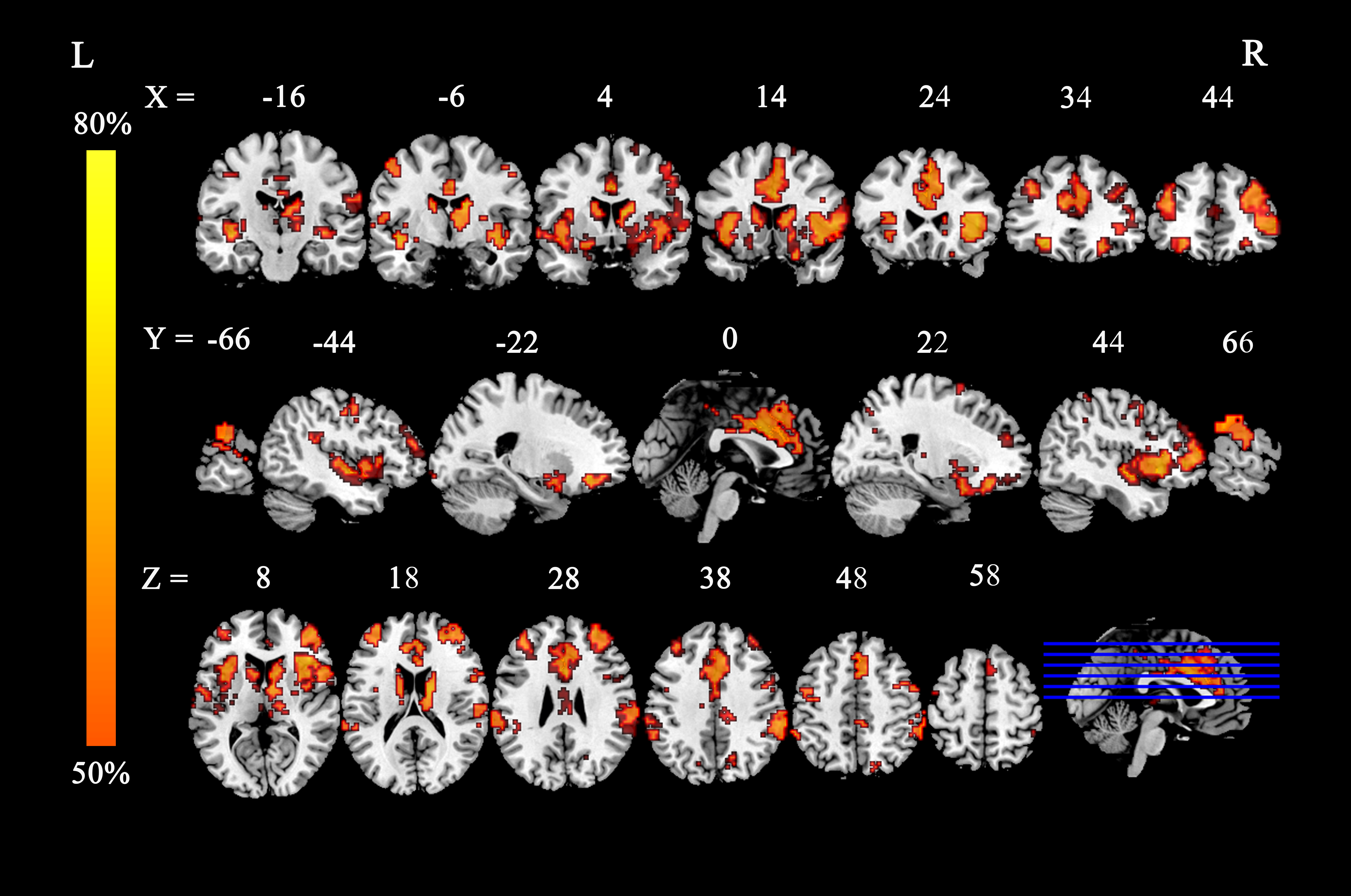

Supplement: SUPPLEMENTARY FIGURE S2 — Smoking-related GM alteration networks based on 1-mm radius sphere. Smoking-related GM alteration networks are shown as network probability maps thresholded at 50%, showing brain regions functionally connected to more than 50% of the contrast seeds. [file Image_2.tif]

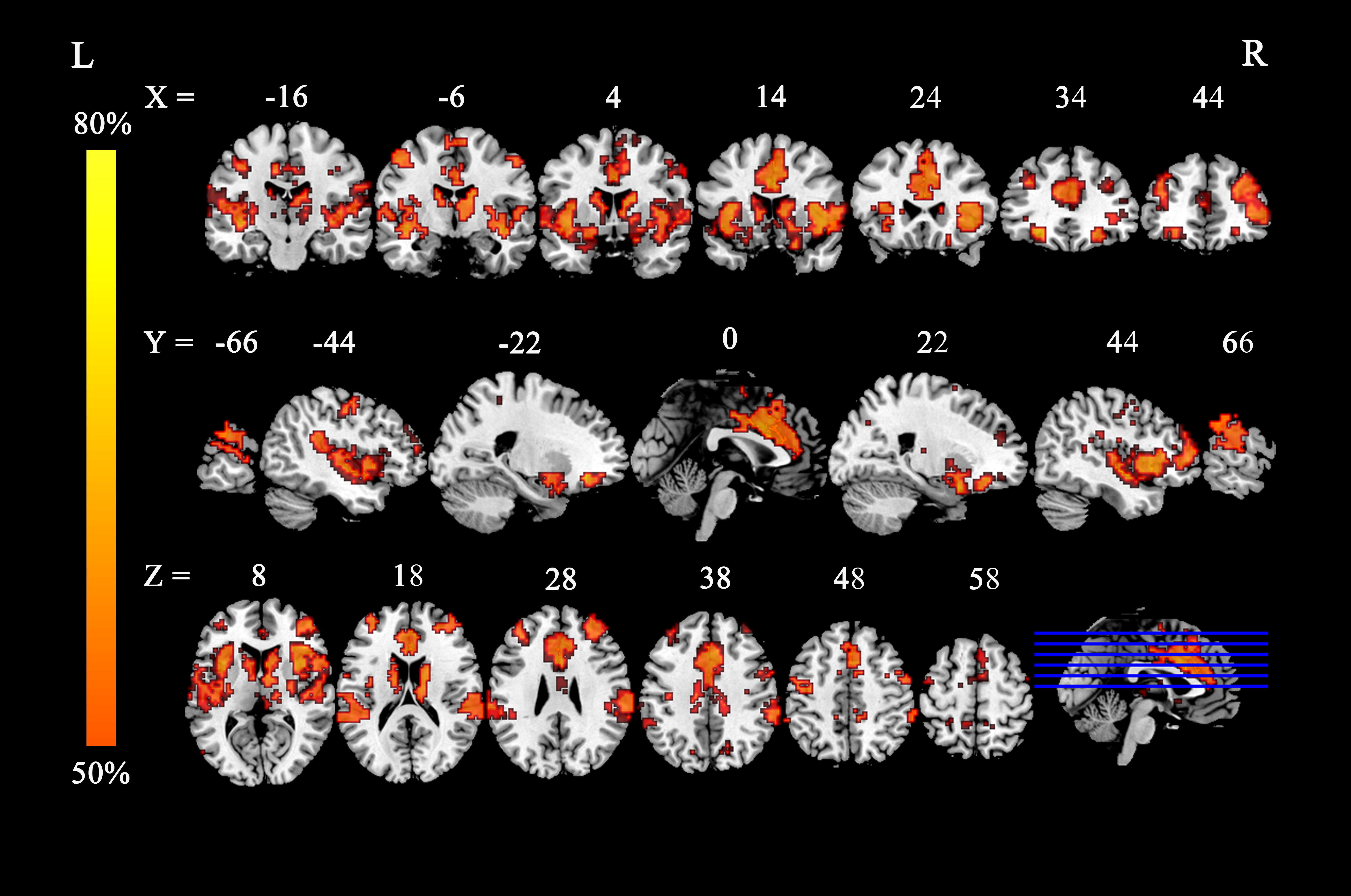

Supplement: SUPPLEMENTARY FIGURE S3 — Smoking-related GM alteration networks based on 7-mm radius sphere. Smoking-related GM alteration networks are shown as network probability maps thresholded at 50%, showing brain regions functionally connected to more than 50% of the contrast seeds. [file Image_3.tif]

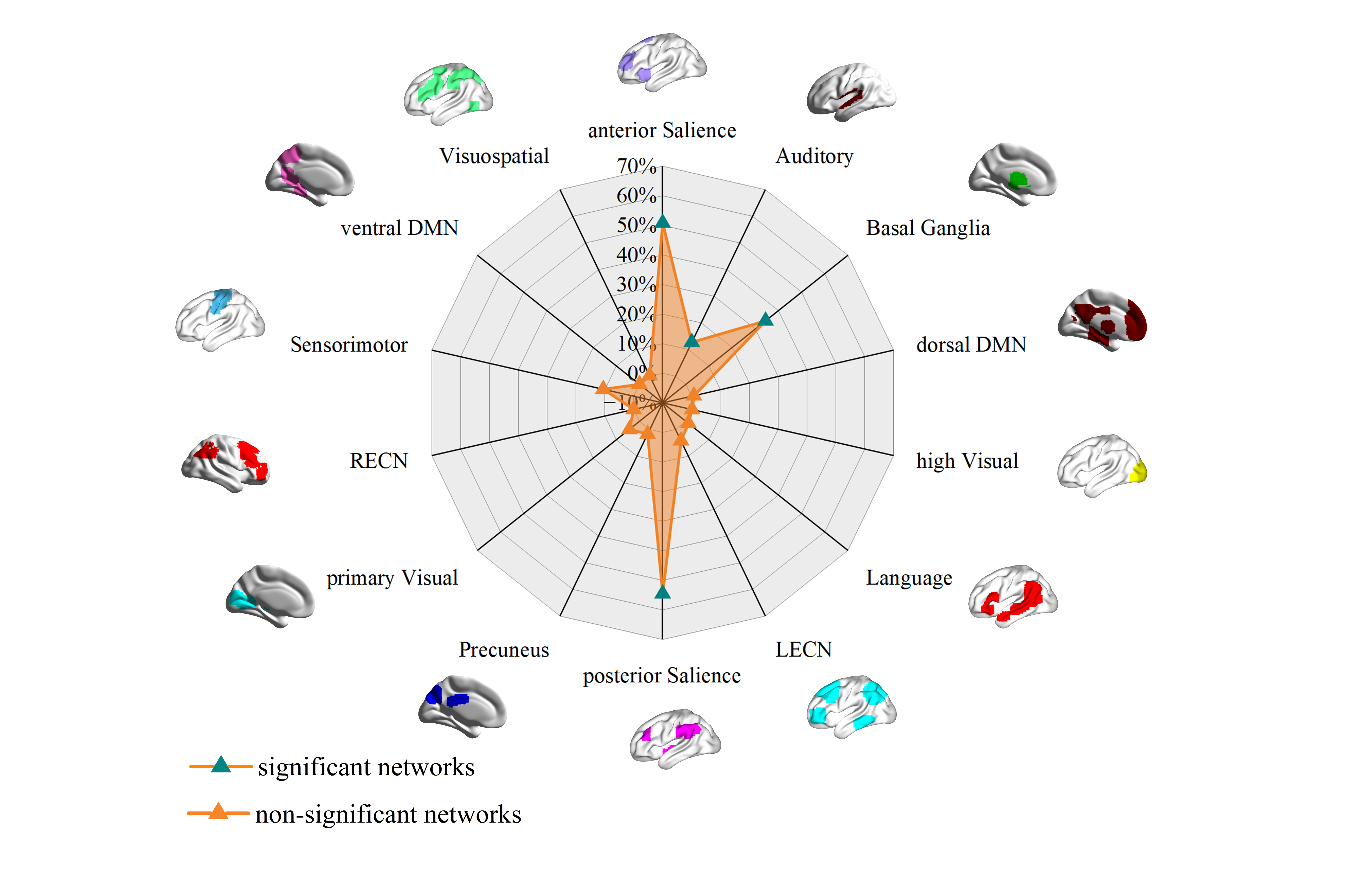

Supplement: SUPPLEMENTARY FIGURE S4 — Associations of GM volume alteration networks with canonical brain networks in smokers based on 1-mm radius sphere. Polar plots display the proportion of overlapping voxels between each GM volume alteration network and a canonical network relative to all voxels within the corresponding canonical network. The green triangles represent GM volume alteration networks, defined as significant networks, exhibiting ≥ 10% overlap with canonical networks, whereas the orange triangles represent non-significant networks with <10% overlap. [file Image_4.tif]

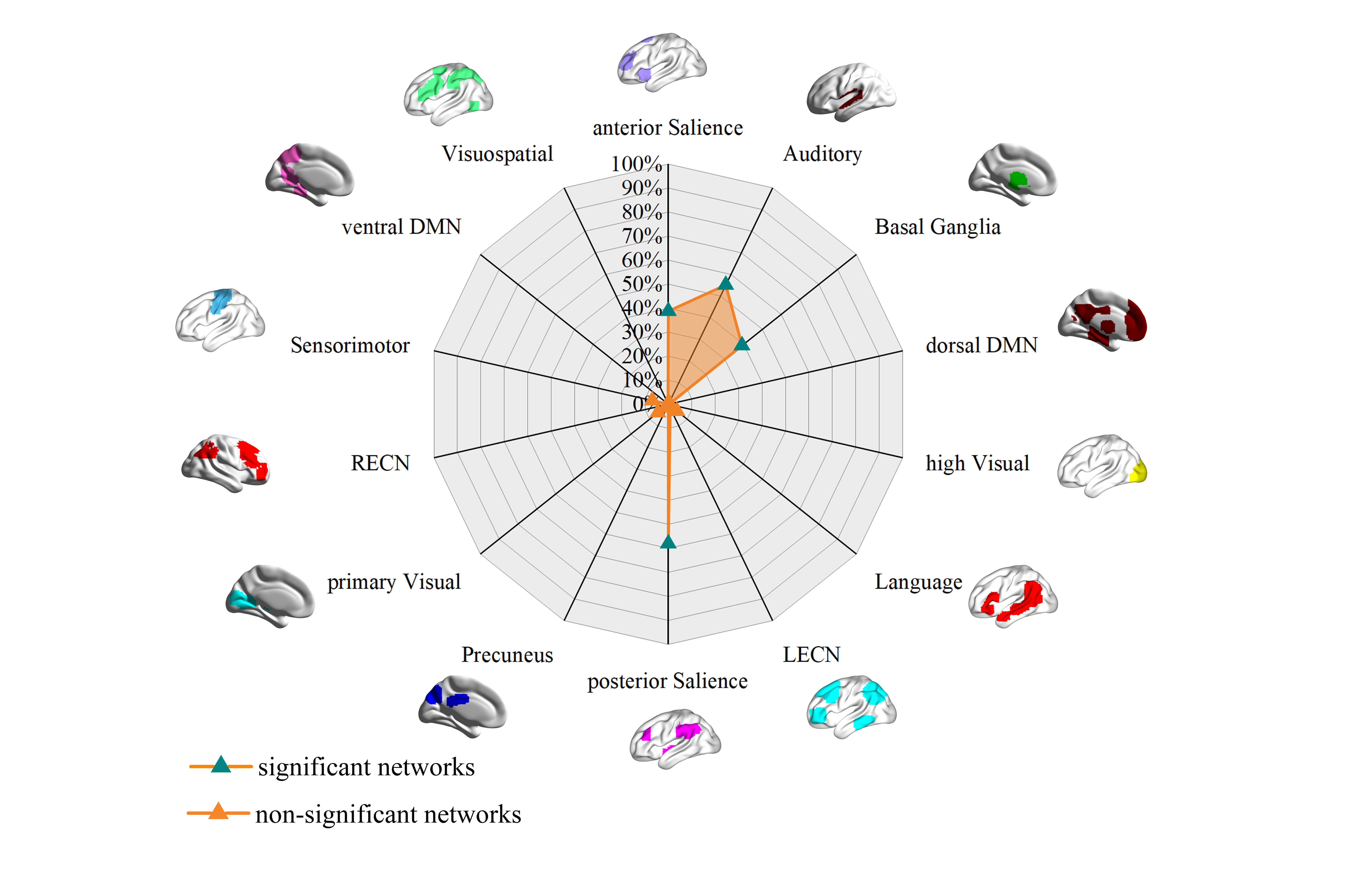

Supplement: SUPPLEMENTARY FIGURE S5 — Associations of GM volume alteration networks with canonical brain networks in smokers based on 7-mm radius sphere. Polar plots display the proportion of overlapping voxels between each GM volume alteration network and a canonical network relative to all voxels within the corresponding canonical network. The green triangles represent GM volume alteration networks, defined as significant networks, exhibiting ≥ 10% overlap with canonical networks, whereas the orange triangles represent non-significant networks with <10% overlap. [file Image_5.tif]

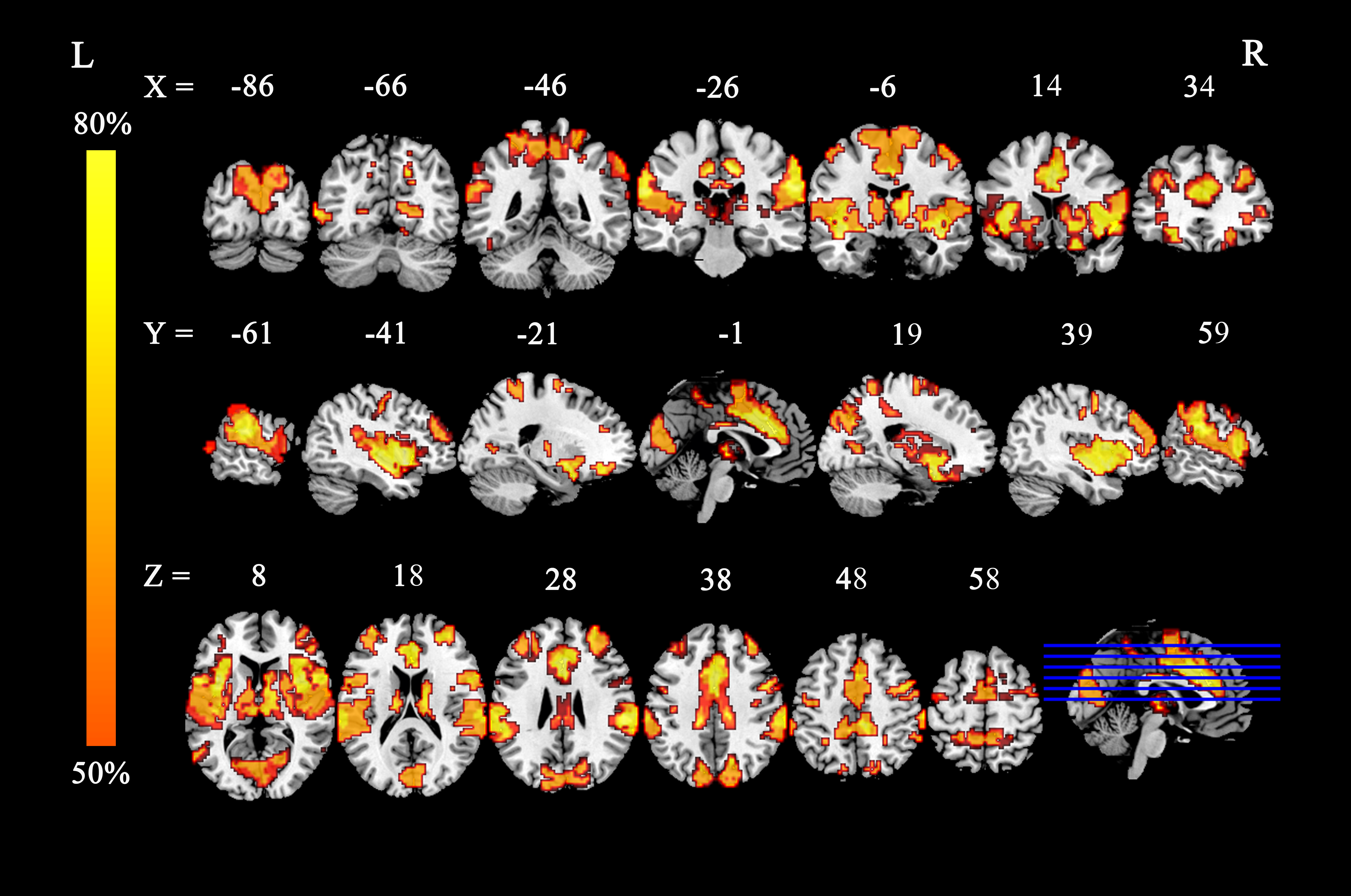

Supplement: SUPPLEMENTARY FIGURE S6 — Smoking-related GM alteration networks in the higher-exposure subgroup based on a 1-mm radius sphere. Smoking-related GM alteration networks are shown as network probability maps thresholded at 60%, showing brain regions functionally connected to more than 60% of the contrast seeds. [file Image_6.tif]

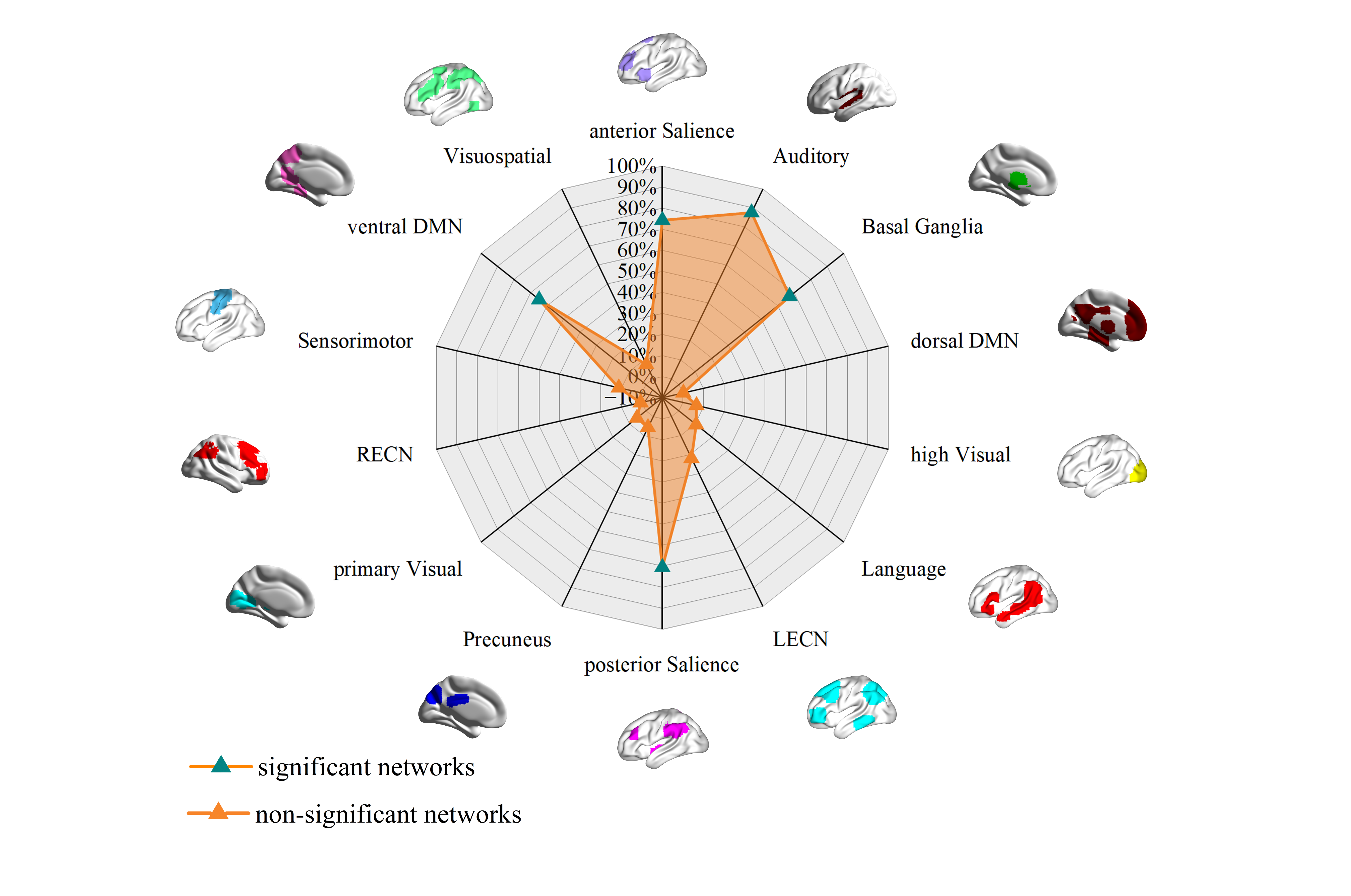

Supplement: SUPPLEMENTARY FIGURE S7 — Associations of GM volume alteration networks with canonical brain networks in the higher-exposure subgroup based on a 1-mm radius sphere. Polar plots display the proportion of overlapping voxels between each GM volume alteration network and a canonical network relative to all voxels within the corresponding canonical network. The green triangles represent GM volume alteration networks, defined as significant networks, exhibiting ≥ 22% overlap with canonical networks, whereas the orange triangles represent non-significant networks with <22% overlap. [file Image_7.tif]

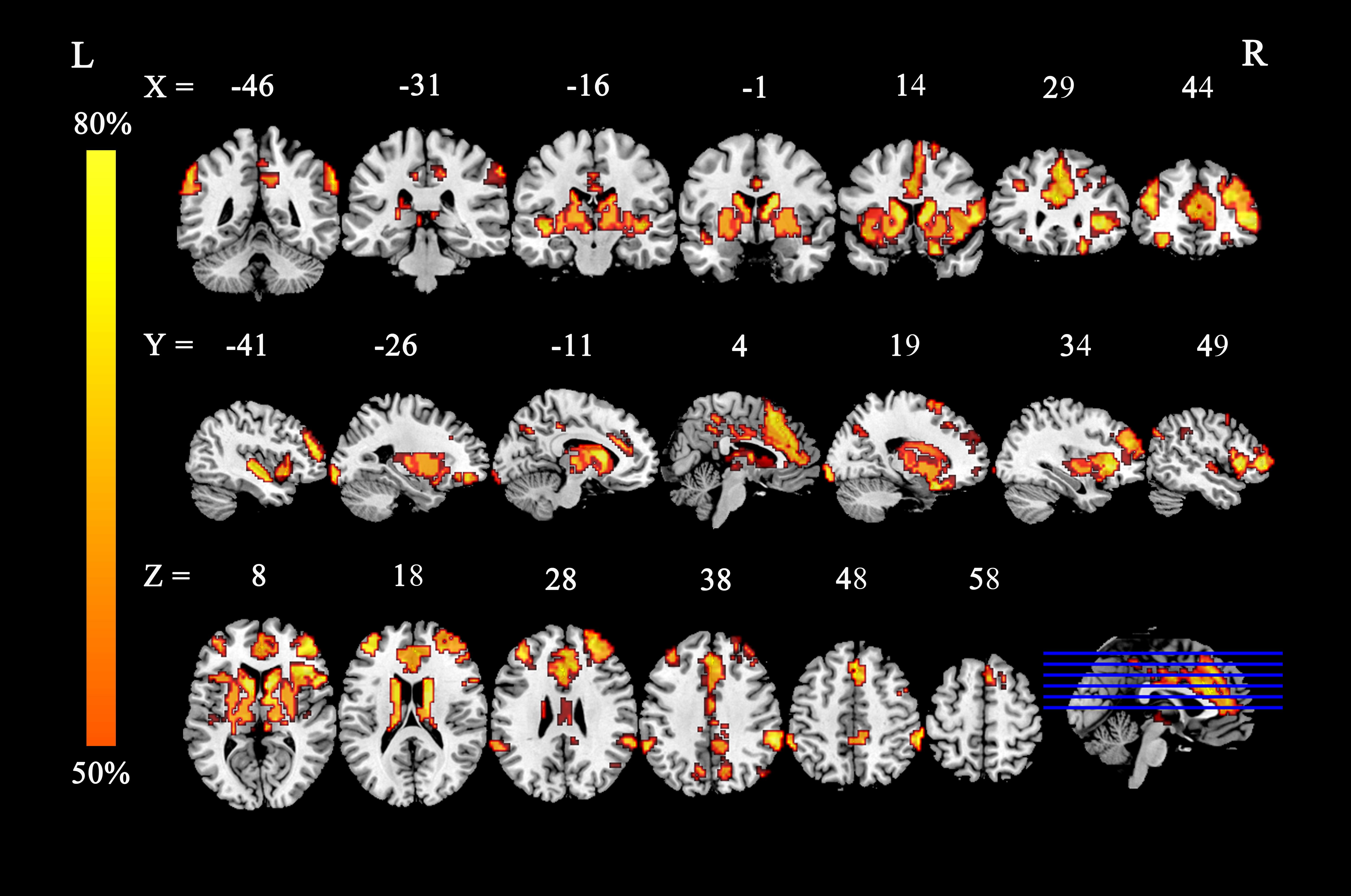

Supplement: SUPPLEMENTARY FIGURE S8 — Smoking-related GM alteration networks in the lower-exposure subgroup based on a 1-mm radius sphere. Smoking-related GM alteration networks are shown as network probability maps thresholded at 60%, showing brain regions functionally connected to more than 60% of the contrast seeds. [file Image_8.tif]

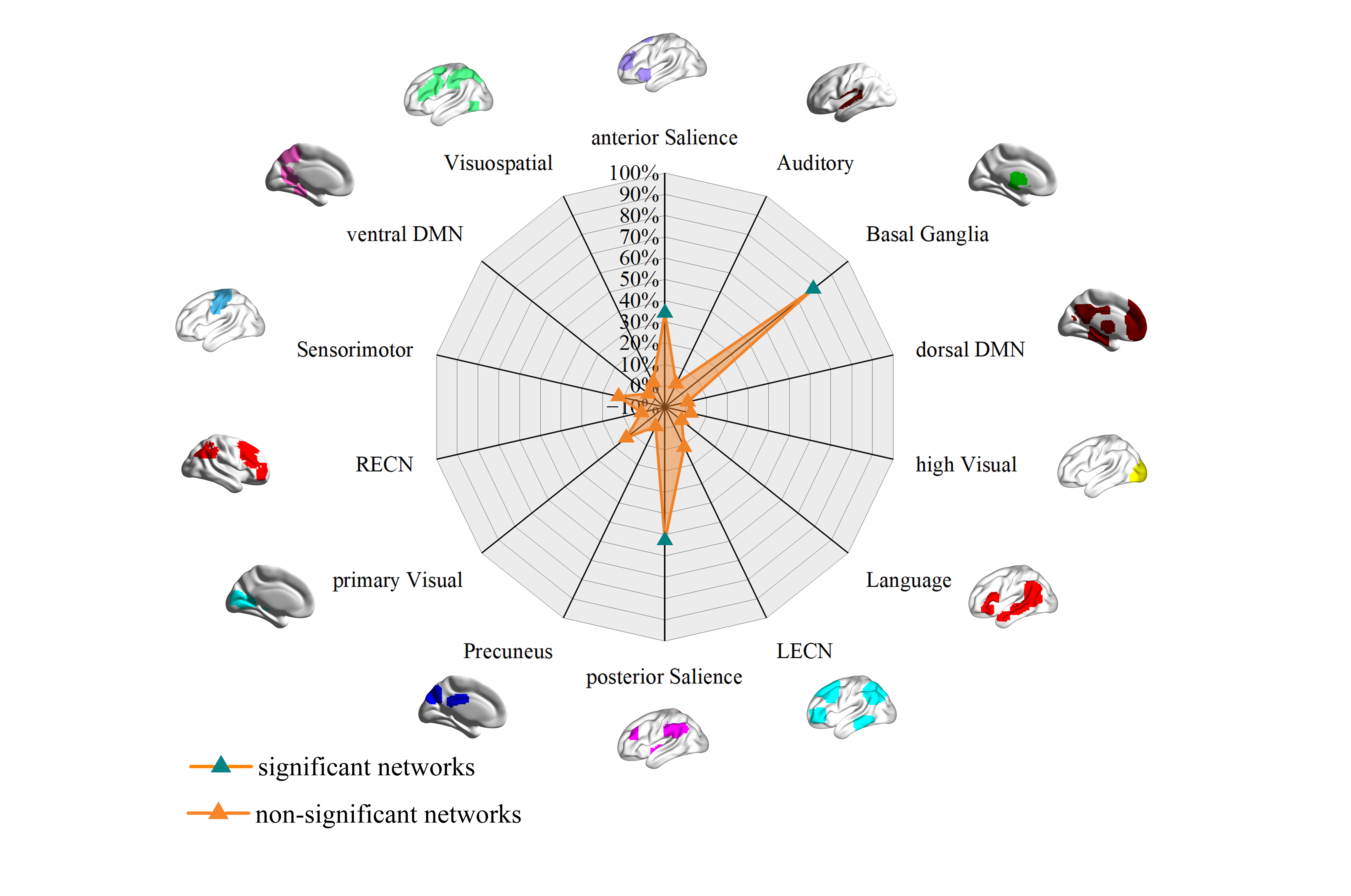

Supplement: SUPPLEMENTARY FIGURE S9 — Associations of GM volume alteration networks with canonical brain networks in the lower-exposure subgroup based on a 1-mm radius sphere. Polar plots display the proportion of overlapping voxels between each GM volume alteration network and a canonical network relative to all voxels within the corresponding canonical network. The green triangles represent GM volume alteration networks, defined as significant networks, exhibiting ≥ 15% overlap with canonical networks, whereas the orange triangles represent non-significant networks with <15% overlap. [file Image_9.tif]

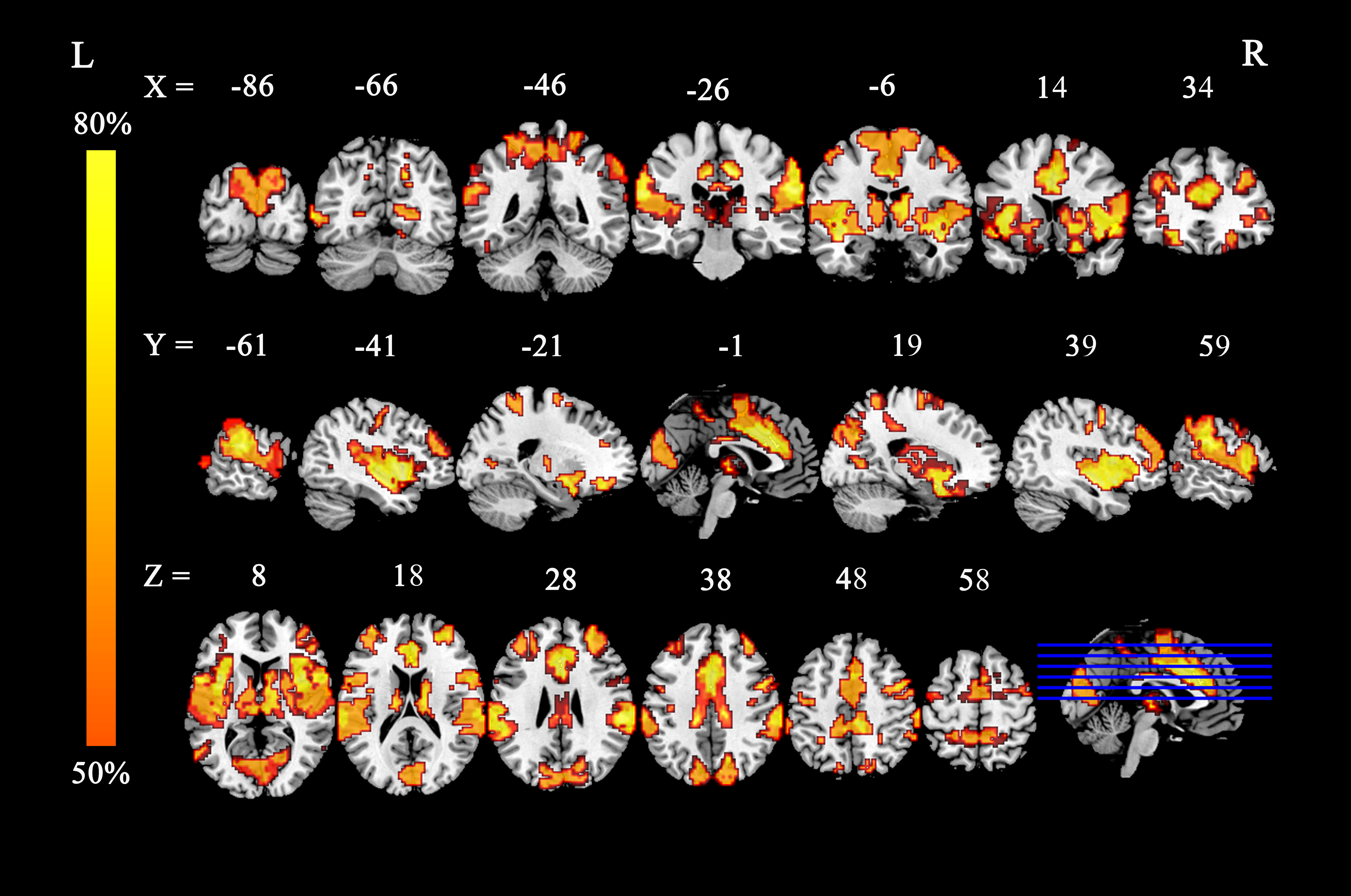

Supplement: SUPPLEMENTARY FIGURE S10 — Smoking-related GM alteration networks in the higher-exposure subgroup based on a 7-mm radius sphere. Smoking-related GM alteration networks are shown as network probability maps thresholded at 60%, showing brain regions functionally connected to more than 60% of the contrast seeds. [file Image_10.tif]

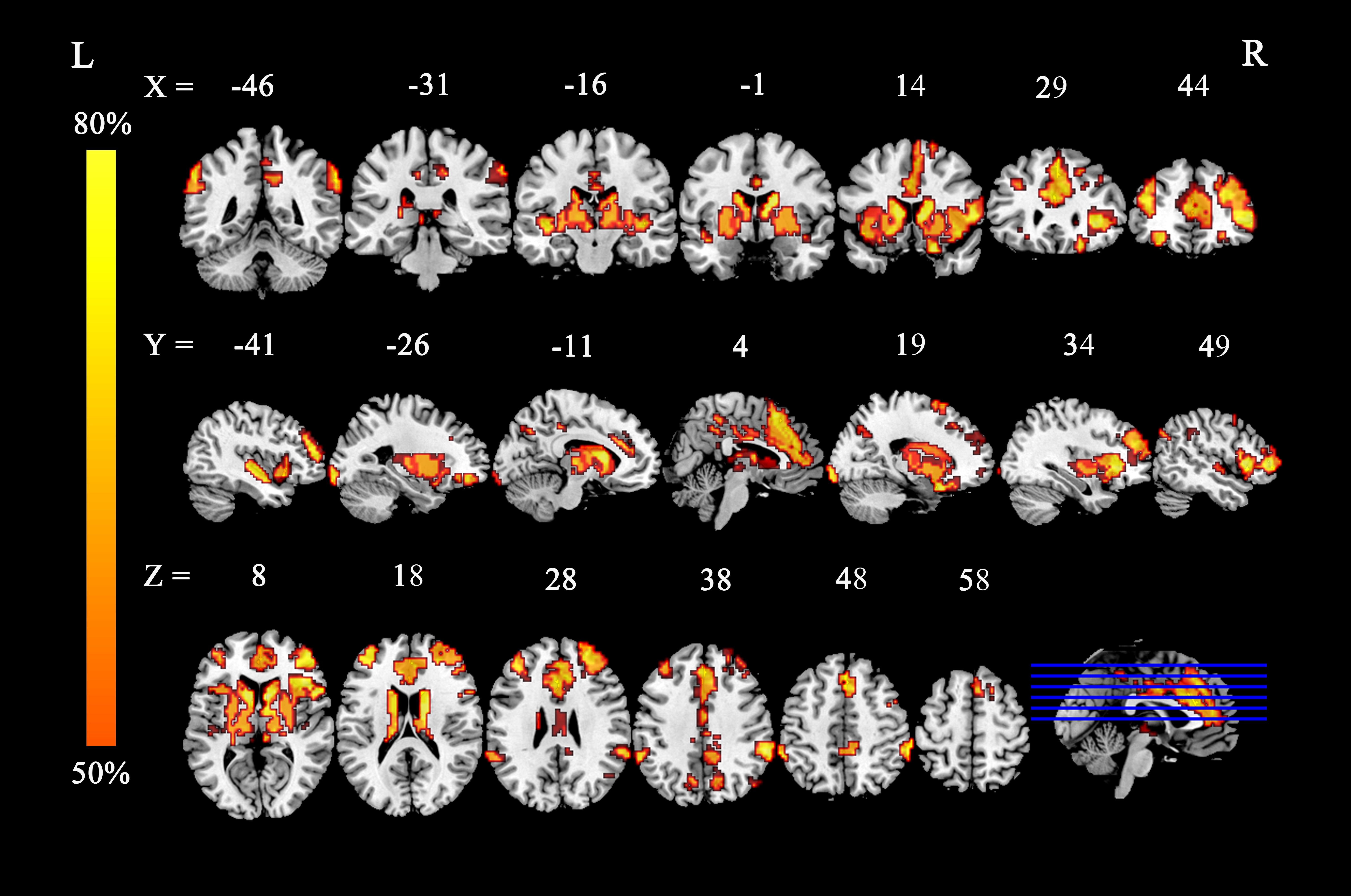

Supplement: SUPPLEMENTARY FIGURE S12 — Smoking-related GM alteration networks in the lower-exposure subgroup based on a 7-mm radius sphere. Smoking-related GM alteration networks are shown as network probability maps thresholded at 60%, showing brain regions functionally connected to more than 60% of the contrast seeds. [file Image_12.tif]
