# Supplementary material for: Network localization of gray matter alterations in chronic smokers using the normative functional connectome
Source: Front Public Health. 2026 Mar 27;14:1762620. doi: 10.3389/fpubh.2026.1762620 (PMC13066286; doi:10.3389/fpubh.2026.1762620)
Supplement: Supplementary file 14 [file Table_1.docx]

**Table S1. Demographic information of the HCP.**

| **Dataset** | **sample size** | **Age (years)** | **Gender (F/M)** |
| --- | --- | --- | --- |
| HCP | 1093 | 28.78 ± 3.69 | 594/499 |

Age is expressed as mean ± standard deviation. HCP, Human Connectome Project; F, female; M, male.
