# Supplementary material for: Network localization of gray matter alterations in chronic smokers using the normative functional connectome
Source: Front Public Health. 2026 Mar 27;14:1762620. doi: 10.3389/fpubh.2026.1762620 (PMC13066286; doi:10.3389/fpubh.2026.1762620)
Supplement: Supplementary file 15 [file Table_2.docx]

**Table S2. Resting-state fMRI parameters of the HCP**

| **Parameter** | **HCP** |
| --- | --- |
| Scanner | 3.0T Siemens Trio |
| Sequence | GRE-EPI |
| TR (ms) | 720 |
| TE (ms) | 33.1 |
| FA (°) | 52 |
| FOV (mm²) | 208×180 |
| Matrix size | 104×90 |
| Slice thickness (mm) | 2 |
| Slice gap (mm) | 0 |
| Slices | 72 |
| Time points | 1210 |

HCP, Human Connectome Project; GRE-EPI, gradient-recalled echo-Planar Imaging; FA, flip angle; fMRI, functional magnetic resonance imaging; FOV, field of view; TE, echo time; TR, repetition time; °, degrees; ms, millisecond.
