# Supplementary material for: Network localization of gray matter alterations in chronic smokers using the normative functional connectome
Source: Front Public Health. 2026 Mar 27;14:1762620. doi: 10.3389/fpubh.2026.1762620 (PMC13066286; doi:10.3389/fpubh.2026.1762620)
Supplement: Supplementary file 16 [file Table_3.docx]

**Table S3. Demographic and clinical characteristics, and technical information of VBM studies included in the higher-exposure chronic smoking subgroup.**

| **Study** | **Sample (female)** | **Age (SD)** | **Smoking History (SD)** | **Cigarette/ day (SD)** | **Pack-years** | **FTND** | **Threshold** | **Software** |
| --- | --- | --- | --- | --- | --- | --- | --- | --- |
| Fritz et al.,  (2014) (1) | CS315 (167)  NS659 (416) | 44.10 (11.84)  51.49 (14.45) | 26.8 (Na) | 13.17 (6.99) | 17.81 (12.25) | Na | p < 0.05 corrected | SPM8 |
| Gallinat et al., (2006) (2) | CS22 (12)  NS23 (12) | 30.8 (7.5)  30.3 (7.9) | 13.9 (7.3) | 14.5 (9.2) | 13.5 (13.0) | 2.9 (1.7) | p < 0.05 corrected | SPM2 |
| Hanlon et al.,  (2016) (3) | CS58 (25)  NS60 (27) | 31.7 (Na)  29.7 (Na) | 15.5 (Na) | 16.2 (Na) | 12.2 (Na) | 4.6 (Na) | p < 0.01 corrected | SPM8 |
| Morales et al., (2012) (4) | CS25 (13)  NS18 (8) | 35.4 (1.8)  30.1 (2.2) | 19 (Na) | 14.1 (1.2) | 11.5 (1.9) | 3.8 (0.4) | p < 0.05 corrected | SPM8 |
| Peng et al.,  (2015) (5) | CS27 (0)  NS53 (0) | 32.26 (3.73)  30.83 (5.18) | Na | 38.70 (8.36) | 31.06 (7.40) | Na | p < 0.05 corrected | SPM8 |
| Shen et al.,  (2018) (6) | CS85 (0)  NS41 (0) | 38.24 (6.81)  38.46 (8.60) | 17.36 (6.58) | 23.46 (9.53) | 20.63 (12.28) | 5.18 (2.18) | p < 0.05 corrected | SPM8 |
| Stoeckel et al., (2016) (7) | CS16 (4)  NS16 (5) | 37.94 (11.61)  34.19 (7.20) | 17.63 (10.49) | 16.00 (4.84) | 16.09 (12.17) | 4.44 (2.16) | p < 0.05 corrected | SPM8 |
| Zhang et al., (2011) (8) | CS48 (24)  NS48 (24) | 31.4 (8.1)  31.1 (8.8) | 12.8 (7.4) | 20.19 (6.6) | 12.9 (7.9) | 5.4 (1.9) | p < 0.05 corrected | FSL |

VBM: voxel-based morphometry; Na: not available; FTND: Fagerstrom test of nicotine dependence; CS: chronic smokers; NS: non-smokers; SPM: statistical parametric mapping; FSL, functional magnetic resonance imaging of the brain software library; FWHM: full width at half maximum.

1. Fritz HC, Wittfeld K, Schmidt CO, Domin M, Grabe HJ, Hegenscheid K, et al. Current smoking and reduced gray matter volume-a voxel-based morphometry study. *Neuropsychopharmacology*. (2014) 39:2594-600. doi: 10.1038/npp.2014.112

2. Gallinat J, Meisenzahl E, Jacobsen LK, Kalus P, Bierbrauer J, Kienast T, et al. Smoking and structural brain deficits: a volumetric MR investigation. *Eur J Neurosci*. (2006) 24:1744-50. doi: 10.1111/j.1460-9568.2006.05050.x

3. Hanlon CA, Owens MM, Joseph JE, Zhu X, George MS, Brady KT, et al. Lower subcortical gray matter volume in both younger smokers and established smokers relative to non-smokers. *Addict Biol*. (2016) 21:185-95. doi: 10.1111/adb.12171

4. Morales AM, Lee B, Hellemann G, O'Neill J, London ED. Gray-matter volume in methamphetamine dependence: Cigarette smoking and changes with abstinence from methamphetamine. *Drug and Alcohol Dependence*. (2012) 125:230-8. doi: 10.1016/j.drugalcdep.2012.02.017

5. Peng P, Wang Z, Jiang T, Chu S, Wang S, Xiao D. Brain-volume changes in young and middle-aged smokers: a DARTEL-based voxel-based morphometry study. *Clin Respir J*. (2017) 11:621-31. doi: 10.1111/crj.12393

6. Shen Z, Huang P, Wang C, Qian W, Yang Y, Zhang M. Cerebellar gray matter reductions associate with decreased functional connectivity in nicotine-dependent individuals. *Nicotine and Tobacco Research*. (2018) 20:440-7. doi: 10.1093/ntr/ntx168

7. Stoeckel LE, Chai XJ, Zhang J, Whitfield-Gabrieli S, Evins AE. Lower gray matter density and functional connectivity in the anterior insula in smokers compared with never smokers. *Addict Biol*. (2016) 21:972-81. doi: 10.1111/adb.12262

8. Zhang X, Salmeron BJ, Ross TJ, Geng X, Yang Y, Stein EA. Factors underlying prefrontal and insula structural alterations in smokers. *Neuroimage*. (2011) 54:42-8. doi: 10.1016/j.neuroimage.2010.08.008
