# Supplementary material for: Network localization of gray matter alterations in chronic smokers using the normative functional connectome
Source: Front Public Health. 2026 Mar 27;14:1762620. doi: 10.3389/fpubh.2026.1762620 (PMC13066286; doi:10.3389/fpubh.2026.1762620)
Supplement: Supplementary file 17 [file Table_4.docx]

**Table S4. Demographic and clinical characteristics, and technical information of VBM studies included in the lower-exposure chronic smoking subgroup.**

| **Study** | | **Sample (female)** | | **Age (SD)** | | **Smoking History (SD)** | | **Cigarette/ day (SD)** | **Pack-years** | | **FTND** | **Threshold** | | | **Software** | |
| --- | --- | --- | --- | --- | --- | --- | --- | --- | --- | --- | --- | --- | --- | --- | --- | --- |
| Bu et al.,  (2016) (1) | | CS26 (0)  NS26 (0) | | 21.42 (1.73)  20.58 (1.47) | | 4.27 (2.44) | | 15.04 (4.82) | 3.55 (2.97) | | 4.42 (2.20) | p < 0.05 corrected | | | SPM8 | |
| Conti et al.,  (2021) (2) | | CS28 (10)  NS24 (11) | | 28.1 (8.3)  28.5 (9.5) | | Na | | 15.0 (4.5) | 10.4 (8.1) | | 5.0 (1.5) | p < 0.05 corrected | | | SPM12 | |
| Daniju et al.,  (2022) (3) | CS19 (14)  NS35 (20) | | 22.8 (3.6)  22.8 (4.9) | | 6.2 (4.2) | | 6.6 (5.3) | | 2.7 (3.65) | Na | | | p < 0.05 corrected | SPM12 | |  |
| Faulkner et al.,  (2021) (4) | | CS12 (8)  NS26 (15) | | 25.40 (4.58)  22.87 (4.60) | | Na | | 11.45 (4.73) | 6.21 (5.37) | | Na | p < 0.001 corrected | | | SPM12 | |
| Franklin et al., (2014) (5) | | CS80 (39)  NS80 (39) | | 33.8 (Na)  22.1 (Na) | | 14.1 (Na) | | 14.7 (Na) | 10.5 (Na) | | 4.45 (Na) | p < 0.025 corrected | | | SPM8 | |
| Peng et al.,  (2015) (6) | | CS26 (0)  NS53 (0) | | 29.42 (4.43)  30.83 (5.18) | | Na | | 16.15 (5.16) | 8.77 (3.57) | | Na | p < 0.05 corrected | | | SPM8 | |
| Wang et al.,  (2014) (7) | | CS22 (0)  NS20 (0) | | 22.48 (2.48)  21.80 (1.32) | | 4.95 (2.27) | | 11.90 (6.13) | 3.10 (2.63) | | Na | p < 0.05 corrected | | | SPM8 | |
| Zhang et al., (2022) (8) | | CS28 (Na)  NS28 (Na) | | 31.29 (5.56)  31.68 (6.57) | | 11.82 (5.77) | | 16.11 (8.35) | 10.08 (7.95) | | 3.54 (2.03) | p < 0.001 corrected | | | SPM12 | |

VBM: voxel-based morphometry; Na: not available; FTND: Fagerstrom test of nicotine dependence; CS: chronic smokers; NS: non-smokers; SPM: statistical parametric mapping; FWHM: full width at half maximum.

1. Bu L, Yu D, Su S, Ma Y, von Deneen KM, Luo L, et al. Functional Connectivity Abnormalities of Brain Regions with Structural Deficits in Young Adult Male Smokers. *Front Hum Neurosci*. (2016) 10:494. doi: 10.3389/fnhum.2016.00494

2. Conti AA, Baldacchino AM. Neuroanatomical Correlates of Impulsive Choices and Risky Decision Making in Young Chronic Tobacco Smokers: A Voxel-Based Morphometry Study. *Front Psychiatry*. (2021) 12:708925. doi: 10.3389/fpsyt.2021.708925

3. Daniju Y, Faulkner P, Brandt K, Allen P. Prefrontal cortex and putamen grey matter alterations in cannabis and tobacco users. *J Psychopharmacol*. (2022) 36:1315-23. doi: 10.1177/02698811221117523

4. Faulkner P, Lucini Paioni S, Kozhuharova P, Orlov N, Lythgoe DJ, Daniju Y, et al. Daily and intermittent smoking are associated with low prefrontal volume and low concentrations of prefrontal glutamate, creatine, myo-inositol, and N-acetylaspartate. *Addict Biol*. (2021) 26:e12986. doi: 10.1111/adb.12986

5. Franklin TR, Wetherill RR, Jagannathan K, Johnson B, Mumma J, Hager N, et al. The effects of chronic cigarette smoking on gray matter volume: influence of sex. *PLoS One*. (2014) 9:e104102. doi: 10.1371/journal.pone.0104102

6. Peng P, Wang Z, Jiang T, Chu S, Wang S, Xiao D. Brain-volume changes in young and middle-aged smokers: a DARTEL-based voxel-based morphometry study. *Clin Respir J*. (2017) 11:621-31. doi: 10.1111/crj.12393

7. Wang K, Yang J, Zhang S, Wei D, Hao X, Tu S, et al. The neural mechanisms underlying the acute effect of cigarette smoking on chronic smokers. *PLoS One*. (2014) 9:e102828. doi: 10.1371/journal.pone.0102828

8. Zhang M, Gao X, Yang Z, Niu X, Wang W, Han S, et al. Integrative brain structural and molecular analyses of interaction between tobacco use disorder and overweight among male adults. *J Neurosci Res*. (2022) 101:232-44. doi: 10.1002/jnr.25141
